# When metabolic enzymes meet lactylation: a bidirectional dialogue in health and disease

**DOI:** 10.3389/fcell.2026.1942974

**Published:** 2026-09-02

**Authors:** Xiang Zhao, Jia-Hui Liu, Kun-Yao Liu, Guo-Chao Lin, Xiao Liu, Zhen Wang, Xin Zhou

**Affiliations:** 1 Cancer Center, The First Hospital of Jilin University, Laboratory of Organ Regeneration and Transplantation of the Ministry of Education, China-Singapore Belt and Road Joint Laboratory on Liver Disease Research, The First Hospital of Jilin University, Changchun, China; 2 Cancer Research Institute of Jilin University, The First Hospital of Jilin University, Changchun, China; 3 International Center of Future Science, Jilin University, Changchun, China; 4 School of Medicine, Hebei University of Engineering, Handan, China

**Keywords:** lactylation, metabolic enzymes, metabolism, lactate, interplay

## Abstract

Lactate accumulation occurs in diverse physiological and pathological states, including hypoxia, ischemia, development, wound healing, immune activation, and cancer, where it functions as a key regulator of cellular energy metabolism, signal transduction, microenvironmental homeostasis, and immune responses. Protein lactylation was first described in 2019, revealing that lactate can serve as a substrate for post-translational protein modification. This review focuses on the bidirectional interplay between metabolic enzymes and lactylation. On one hand, metabolic enzymes control lactate production and thereby shape the lactylation landscape. On the other hand, lactylation exerts feedback regulation through two mechanisms: directly, lactylation of metabolic enzymes *per se* alters enzyme properties, including activity, stability, and interactions; indirectly, histone lactylation epigenetically modulates the expression of metabolic enzymes, and the lactylation of other proteins alters the expression or activity of metabolic enzymes. Through this integrated framework, we aim to elucidate how lactylation influences the functional traits of metabolic enzymes and how lactylation of metabolic enzymes, in turn, affects disease onset and progression. Finally, we explore whether targeting lactylation of metabolic enzymes could emerge as a promising therapeutic avenue.

## 1 Introduction

Lactic acid was first discovered in 1780 by Carl Wilhelm Scheele in sour milk (Kompanje et al., 2007). It is a three-carbon organic acid primarily generated from pyruvate via lactate dehydrogenase (LDH), and is subsequently transported across cell membranes by monocarboxylate transporters (MCTs) (Brooks, 1986). For decades, lactate was long regarded as a metabolic waste (Gladden, 2004). However, emerging evidence over the past two decades has reshaped this view, revealing that lactate serves as a critical energy substrate, a gluconeogenic precursor, a cellular redox buffer, a metabolic signaling molecule, an epigenetic regulator, and a modulator of immune and inflammatory responses (Hui et al., 2017; Li et al., 2022; Yang et al., 2022; Hensel et al., 2025; Llibre et al., 2025). Despite this progress, the molecular mechanisms underlying lactate’s other functions remain largely unclear.

The discovery of lysine lactylation (Kla) in 2019 provided a pivotal mechanistic breakthrough (Zhang et al., 2019). Identified initially on histones, lactylation was shown to regulate gene transcription. Since then, a growing number of “writers,” “erasers,” and potential “readers” of lactylation have been characterized (Zong et al., 2025; Sheng et al., 2026). Beyond histones, lactylation has been found to occur on non-histone proteins, where it regulates diverse protein properties including enzymatic activity, stability, subcellular localization, and interactions with other molecules (Zhang et al., 2023; Huang Y. et al., 2024; Meng et al., 2024a; Zeng et al., 2025; Huang et al., 2026). These findings position lactylation as a novel post-translational modification linking lactate to diverse physiopathological processes, such as exercise, neuronal activity, tissue injury, infection, inflammation, aging, cancer, metabolic disorders, cardiovascular diseases, and degenerative diseases (Han et al., 2023; Hu Y. et al., 2024; Qiao et al., 2024; Yan et al., 2024; Zhu et al., 2024; Liu et al., 2025d; Deng et al., 2026).

Lactylation is a distinctive post-translational modification (PTM) among the expanding repertoire of protein modifications, as it directly links intracellular lactate metabolism with protein regulation. In turn, lactylation regulates metabolism by modulating metabolic enzyme activity, metabolic gene expression, and metabolism-related signaling, forming a reciprocal regulatory loop (Liu J. et al., 2025; Huang et al., 2026; Li J. et al., 2026).

**FIGURE 1:**
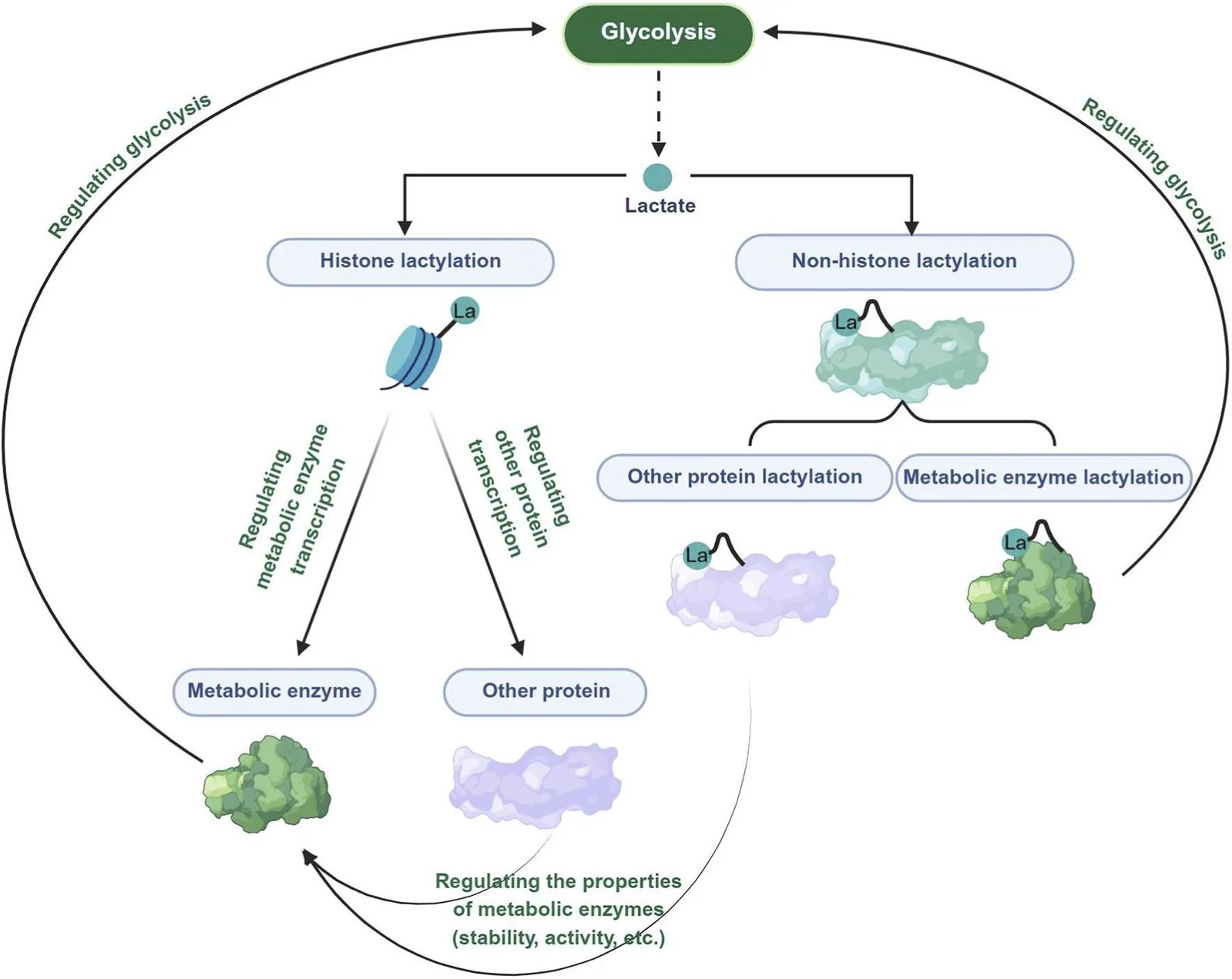
Lactylation-mediated feedback regulatory loop of glycolysis. Glycolysis drives lactate production, which in turn promotes the lactylation of both histone and non-histone proteins. Histone lactylation modulates the transcription of metabolic enzymes and other proteins, whereas non-histone lactylation can be divided into lactylation of metabolic enzymes and lactylation of other proteins. Histone lactylation regulates the expression levels of various proteins, which, together with the lactylation of non-metabolic enzymes, coordinately influences the abundance and activity of metabolic enzymes, thereby feeding back to regulate glycolysis. Additionally, direct lactylation of metabolic enzymes can alter their enzymatic activities and functional properties, further modulating glycolytic flux. This multilayered interplay between lactylation and glycolysis constitutes a self-regulating feedback loop. Created with BioRender.com.

Previous reviews have primarily focused on the mechanisms and biological functions of lactylation, or have explored its relationship with metabolic reprogramming from a largely unidirectional perspective (Chen et al., 2021; Liu et al., 2022; Yang J. et al., 2025). In contrast, this review summarizes recent advances in the reciprocal interplay between cellular metabolism and protein lactylation (Figure 1), with a particular focus on metabolic enzymes as both regulators and targets of lactylation. We emphasize how metabolic enzymes influence lactylation through their roles in lactate production, as well as how lactylation, in turn, regulates metabolic enzyme function and alters metabolic pathways. We also discuss the roles of this regulatory network in human diseases, current challenges, future directions, and the therapeutic potential of targeting metabolic enzyme lactylation.

## 2 Lactylation regulation

### 2.1 The discovery of lactylation

**FIGURE 2:**
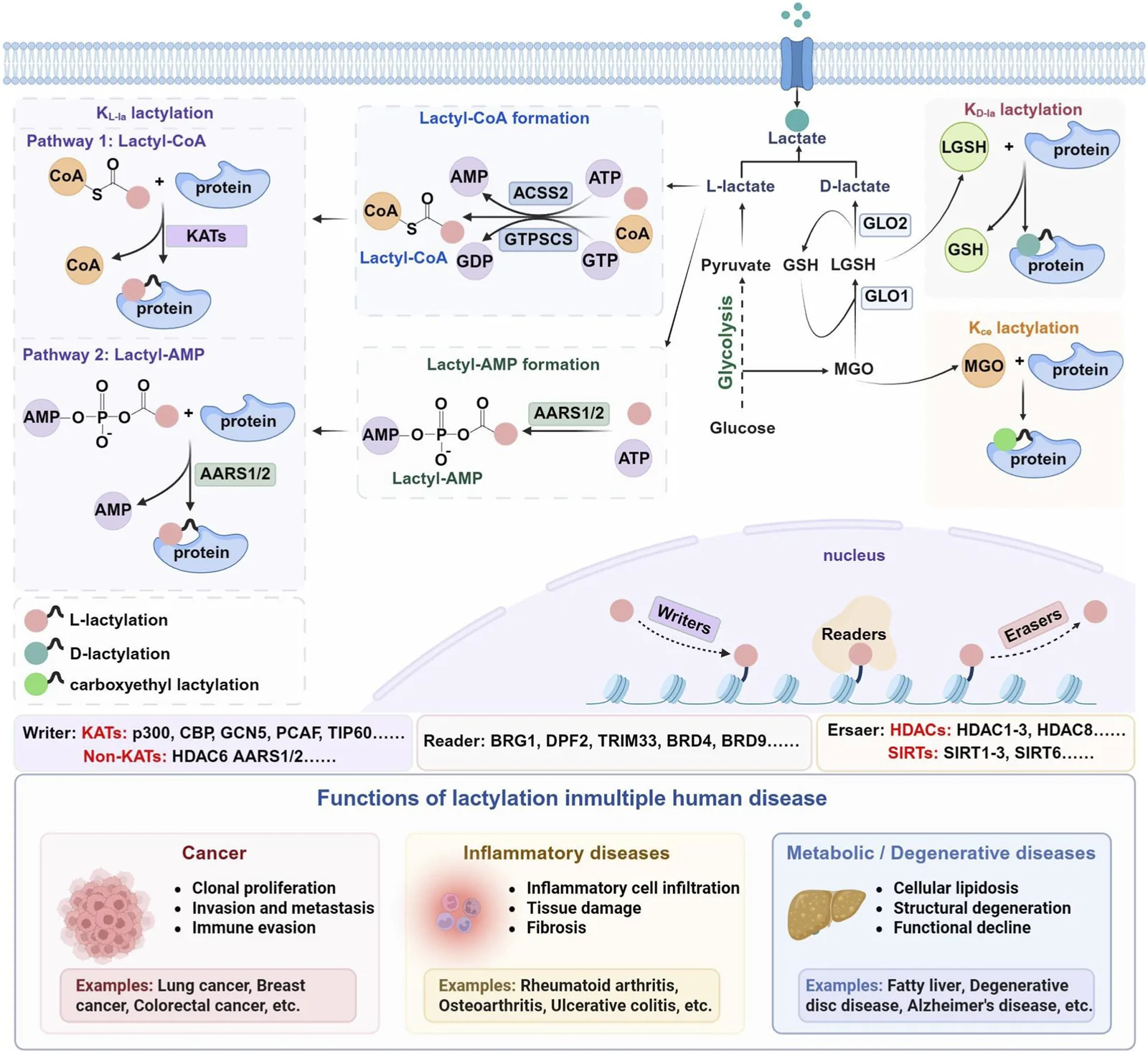
Dynamic regulation of protein lactylation. Protein lactylation is a dynamic, post-translational modification mediated by multiple pathways and tightly regulated by writer, reader, and eraser proteins. The modification primarily originates from two sources: (1) K_L-la_, which is generated via L-lactate-derived pathways, including lactyl-CoA intermediates produced independently by ACSS2 and GTPSCS, as well as lactyl-AMP intermediates generated by AARS1/2. These intermediates are utilized by writers (KATs and AARS1/2, respectively) to catalyze the addition of lactyl groups to target proteins. (2) K_D-la_ and K_ce_, which are generated via the glyoxalase pathway in a writer-independent manner using LGSH and MGO as substrates. Furthermore, the modification process depends on the recognition of specific lactylation sites by readers and the removal of lactyl groups by erasers. Writers, readers, and erasers collectively regulate protein lactylation, thereby influencing epigenetic regulation and gene expression. Created with BioRender.com.

Numerous metabolites serve as donors of PTMs, giving rise to diverse modifications such as acetylation, succinylation, and malonylation. In 2019, a new metabolite-derived modification—lactylation—was discovered by Professor Yingming Zhao’s team, with evidence demonstrating that histone lactylation regulates gene transcription on chromatin (Zhang et al., 2019). Subsequent studies have revealed that protein lactylation primarily occurs on lysine residues and exists as three distinct isomers. The initially identified form, reported by Zhang and colleagues, is lysine L-lactylation (K_L-la_) (Zhang et al., 2019). The other two isomers are lysine D-lactylation (K_D-la_) and N-ε-(carboxyethyl)-lysine (K_ce_) (Zhang D. et al., 2025). L-lactate required for K_L-la_ is produced from pyruvate via lactate dehydrogenase A (LDHA). In contrast, both D-lactate (for K_D-la_) and the carboxyethyl group (for K_ce_) are derived from methylglyoxal (MGO) (Figure 2). K_D-la_ is largely produced via a non-enzymatic reaction between lysine residues and lactoylglutathione (LGSH), which is formed from MGO and glutathione (GSH) through the action of glyoxalase 1 (GLO1) (Gaffney et al., 2020). In contrast, K_ce_ is generated by direct modification of the lysine ε-amine group by MGO (Galligan et al., 2018). The cellular levels of K_ce_ and K_D-la_ are substantially lower and more challenging to detect; therefore, research on these two isomers remains limited. Consequently, the vast majority of studies to date have focused on K_L-la_. Unless otherwise specified, “lactylation” in this review refers exclusively to K_L-la_.

### 2.2 Lactylation writers

#### 2.2.1 Lactyltransferases

Lactylation is catalyzed by two major classes of lactyltransferases: lysine acetyltransferases (KATs/HATs) and alanyl-tRNA synthetases (AARS1/2) (Figure 2).

These KAT family lactyltransferases are believed to utilize lactyl-CoA as a substrate. Through HPLC and mass spectrometry validation, Zhang et al. identified p300 as the first reported lactyltransferase involved in histone lactylation (Zhang et al., 2019). Subsequently, several other KAT family members have been extensively studied, including p300, CREB-binding protein (CBP), general control non-derepressible 5 (GCN5, also known as KAT2A), p300/CBP-associated factor (PCAF), Tat-interactive protein 60 (TIP60, also known as KAT5), histone acetyltransferase binding to ORC1 (HBO1, also known as KAT7), and males absent on the first (MOF, also known as KAT8). These lactyltransferases modify both histones and non-histones. For example, p300 lactylates H4K12 and YTH domain-containing protein 1 (YTHDC1) (Dai et al., 2025; Deng et al., 2025). GCN5 lactylates H3K9 and RAD51 (Sun C. et al., 2025).

In contrast, AARS1/2 generate lactyl-AMP directly from lactate and ATP, which serves as the lactyl donor for protein lactylation, analogous to canonical aminoacylation. AARS1/2 can also lactylate both histones and non-histones, including pyruvate dehydrogenase E1 subunit alpha 1 (PDHA1), carnitine palmitoyltransferase 2 (CPT2), cyclic GMP-AMP synthase (cGAS), and tumor protein p53 (p53) (Ju et al., 2024; Li et al., 2024; Mao et al., 2024; Zong et al., 2024).

Unexpectedly, lactyltransferase activity may also be present within the HDAC family. Histone deacetylase 6 (HDAC6), a deacetylase, was reported to exhibit lactyltransferase activity. Sun et al. demonstrated that HDAC6 catalyzes α-tubulin lactylation at K40 (Sun et al., 2024).

#### 2.2.2 Donor substrates for lactylation

Lactyltransferases require an intermediate to transfer lactate to lysine residues. To date, two such intermediates have been identified: lactyl-CoA and lactyl-AMP (Figure 2).

Lactyl-CoA is generated through two distinct pathways. The first pathway involves acetyl-CoA synthetase 2 (ACSS2) (Figure 2). Zhu et al. reported that extracellular signal-regulated kinase (ERK) -mediated ACSS2 S267 phosphorylation promotes its nuclear translocation, where ACSS2 generates lactyl-CoA from lactate and CoA (Zhu R. et al., 2025). The second pathway relies on guanosine triphosphate-specific succinyl-CoA synthetase (GTPSCS) (Figure 2). This enzyme produces succinyl-CoA or lactyl-CoA depending on substrate availability, predominantly acting as a lactyl-CoA synthetase in the nucleus (Liu R. et al., 2025).

Lactyl-AMP is generated by AARS1/2. Upon lactate activation by AARS, lactate reacts with ATP to form the intermediate lactyl-AMP. This intermediate subsequently transfers the lactyl group to target proteins through the action of AARS1/2 (Figure 2). The initial characterization of AARS proteins in this context came from Professor Shimin Zhao’s team, who discovered that AARS2 functions as a lactyltransferase, enhancing the lactylation of PDHA1 and CPT2 (Mao et al., 2024). Recent studies showed that AARS1/2 also lactylate cGAS, p53, and the YAP–TEAD complex (Ju et al., 2024; Li et al., 2024; Zong et al., 2024).

#### 2.2.3 Non-enzymatic, spontaneous reactions of K_D-la_ and K_ce_

K_D-la_ and K_ce_ are produced through non-enzymatic, spontaneous reactions (Figure 2). LGSH is required as a precursor for K_D-la_. LGSH is produced when MGO is acted upon by the glyoxalase system, as described above. Once LGSH accumulates sufficiently, it spontaneously transfers the lactyl group to lysine residues, releasing GSH (Gaffney et al., 2020). Similarly, K_ce_ is formed non-enzymatically through the direct modification of lysine residues by MGO (Galligan et al., 2018).

### 2.3 Lactylation erasers

PTMs are dynamic and reversible; just as writers add modification groups, erasers remove them (Figure 2). Protein lactylation has dedicated delactylases. Previous studies have shown a significant overlap between lactylation and acetylation sites (Sung et al., 2023). Histone acetyltransferases such as p300 and CBP catalyze both acetylation and lactylation (Zeng et al., 2023). Conversely, deacetylases can function as delactylating enzymes. HDACs and sirtuins (SIRTs) are the major delactylases.

Within the HDAC family, HDAC1-3 and HDAC8 are capable of delactylation. HDAC1-3 can delactylate both K_L-la_ and K_D-la_ (Moreno-Yruela et al., 2022). For example, HDAC1 reduces lactylation of histones near *ATF3*, *ATF4*, and *CHAC1*, suppressing their expression (Zhu et al., 2025a). HDAC3 has been identified as the most efficient delactylase within the HDAC family. It can remove lactyl groups from H3K18la, thereby suppressing breast cancer progression (Moreno-Yruela et al., 2022; Xu Y. et al., 2025). Notably, HDAC1-3 also delactylate non-histones. For example, HDAC2 delactylates N6-methyltransferase-like 3 (METTL3) (He X. et al., 2025). HDAC8 mediates non-histone delactylation, and hypoxia-induced HDAC8 reduction increases protein arginine methyltransferase 1 (PRMT1) K134/K145 lactylation in triple-negative breast cancer (Zhou J. et al., 2025).

The SIRT family also plays a significant role in delactylation. A recent study showed that SIRT1 and SIRT2 exhibit much higher delactylase activity than SIRT3 and SIRT5. SIRT2 acts as an eraser of histone lactylation in neuroblastoma cells, where increased histone lactylation promotes cell proliferation and migration (Zu et al., 2022). In gastric cancer cells, SIRT1 reduces H3K18la, thereby suppressing gastric cancer progression (Tsukihara et al., 2025). SIRT3 delactylates H3K9la and malic enzyme 2 (ME2) K352, suppressing esophageal squamous cancer progression and colorectal cancer growth, respectively (Chen C. et al., 2025; Li C. et al., 2025).

The above discussion raises an important question: how is the specificity of delactylases determined? Different HDAC and SIRT isoforms exhibit distinct subcellular localization patterns, which may influence their substrate accessibility (Michishita et al., 2005; Yang and Seto, 2008). HDAC1–3, HDAC8, SIRT1, and SIRT2 can access the nucleus and regulate nuclear histone and non-histone substrates, whereas SIRT3 is predominantly localized in mitochondria and targets mitochondrial substrates such as ME2 (Moreno-Yruela et al., 2022; Zu et al., 2022; He X. et al., 2025; Li C. et al., 2025; Tsukihara et al., 2025; Xu Y. et al., 2025; Zhou J. et al., 2025; Zhu et al., 2025a). Therefore, subcellular localization may represent an important determinant of delactylase specificity, although other factors, including substrate recognition and protein interactions, may also contribute.

Beyond their canonical deacetylase activity and recognized delactylase function, HDACs and SIRTs exhibit broader lysine deacylase activities, suggesting that these enzymes may participate in the regulation of diverse acylation modifications. For example, HDACs contribute to the removal of lysine crotonylation, whereas SIRT5 functions as a key desuccinylase (Yoshida et al., 2017; Ke et al., 2025).

The landscape of delactylases remains incomplete. Additional delactylases within and beyond the HDAC and SIRT families remain to be identified. The specificity of delactylases requires further investigation.

### 2.4 Lactylation readers

The functions of protein lactylation are primarily mediated by lactylation readers, which recognize specific lactylation sites and regulate downstream signaling or transcription (Figure 2). Lactylation readers, including brahma-related gene 1 (BRG1), tripartite motif-containing protein 33 (TRIM33), BRD4, and BRD9, recognize specific histone lactylation sites through bromodomains, regulating chromatin remodeling, gene transcription, and biological processes (Hu X. et al., 2024; Nunez et al., 2024; Zhang F. et al., 2024; Wei et al., 2026). In addition, double PHD fingers 2 (DPF2) represents another reader protein. It recognizes H3K14la through its DPF domain, promoting chromatin relaxation and gene transcription (Zhai et al., 2024). Collectively, these readers translate histone lactylation into epigenetic regulation.

### 2.5 Functions of lactylation

The effects of lactylation on protein function have been comprehensively reviewed elsewhere (Shi et al., 2025). Here, we summarize its major functional outcomes, including epigenetic regulation, transcription, enzyme activity, protein stability, molecular interactions, and subcellular localization.

#### 2.5.1 Epigenetic regulation

Epigenetic regulation of gene transcription is the best-characterized function of histone lactylation. Histone lactylation alters chromatin structure to regulate gene transcription. For example, p300, the first identified histone lactyltransferase, can lactylate histones H3 and H4, thereby promoting the conversion of M1 macrophages to the M2 phenotype (Zhang et al., 2019). HBO1 lactylates H3K9, thereby promoting gene transcription (Niu et al., 2024). Currently identified histone lactylation sites include H3K23la, H3K27la, H4K12la, H4K79la, and H4K5la, among others (Peng and Du, 2025). Most lactylation sites are located on histones H3 and H4, with a few also identified on H2A and H2B.

#### 2.5.2 Transcription

Lactylation regulates gene transcription not only through histone modification but also via direct modification of transcription factors and cofactors, thereby either promoting or repressing their transcriptional functions. In lung cancer, p53 lactylation at K120 inhibits its transcriptional activity, reducing the expression of apoptosis-related genes such as *PUMA* and *BAX* (Ding et al., 2026). In gallbladder cancer, YY1 lactylation at K183 enhances its DNA-binding activity, activating *FBXO33* transcription and promoting metastasis (Wu Z. et al., 2025). In colorectal cancer, protein arginine methyltransferase 5 (PRMT5) K240 lactylation represses *alkB homolog 5 (ALKBH5)* transcription and promotes ferroptosis resistance (Qu et al., 2025). Collectively, lactylation represents a metabolic state-dependent regulatory mechanism that modulates transcription through transcription factors and epigenetic regulators, rather than acting as an inherently activating or repressive modification.

#### 2.5.3 Enzyme activity

Enzymatic reactions are essential for cellular survival, and lactylation serves as a key modulator of enzyme activity. For instance, GCN5-mediated lactylation of ERK promotes its dimerization and activation, thereby activating the ERK signaling pathway and driving tumor progression (Huang et al., 2026). Lactylation of PDHA1 at K336 inhibits its activity, which in turn suppresses oxidative phosphorylation (OXPHOS) (Mao et al., 2024). Collectively, these examples illustrate that lactylation can either potentiate or inhibit enzyme activity in a context-dependent manner.

#### 2.5.4 Protein stability

Lactylation regulates protein stability in a context-dependent manner, often by inhibiting ubiquitination. For example, transcription factor EB (TFEB) is lactylated by p300 at K91, which prevents its interaction with the E3 ubiquitin ligase WW domain-containing E3 ubiquitin protein ligase 2 (WWP2), thereby inhibiting degradation and promoting lysosomal biogenesis and autophagy (Huang Y. et al., 2024). However, this is not universal. A notable counterexample is cGAS, whose K21 lactylation promotes proteasomal degradation (Rao et al., 2025). Thus, lactylation can either stabilize or destabilize target proteins depending on the specific protein, the modified residue, and the cellular context.

#### 2.5.5 Molecular interactions

Protein lactylation is intimately linked to molecular interactions, including protein-protein interactions, protein-RNA interactions, and protein-metabolite binding. SIRT1-mediated delactylation of α-myosin heavy chain K1897 reduces its interaction with titin, contributing to heart failure (Zhang et al., 2023). Beyond protein-protein interactions, lactylation also affects protein-RNA binding. Methyltransferase-like 16 (METTL16) methylates mRNA to promote translation. Lactylation of METTL16 at K229 enhances its binding to *ferredoxin 1* (*FDX1*) mRNA, facilitating methylation and subsequently increasing the stability and translation efficiency of *FDX1* mRNA, ultimately enhancing FDX1 expression (Sun et al., 2023). In addition, lactylation can disrupt protein-metabolite binding. Elevated lactate induces lactylation of the cluster of differentiation 44 (CD44) on the surface of CD8^+^ T cells, which disrupts CD44 binding to hyaluronan, ultimately suppressing anti-tumor immunity (Yang et al., 2026).

#### 2.5.6 Subcellular localization

Lactylation also regulates the subcellular localization of proteins, thereby orchestrating their context-dependent functions. In lung adenocarcinoma, syrosingopine-induced lactate accumulation promotes RIG-I K518/K657 lactylation, which inhibits PARP1 PARylation and impairs DNA repair. Given that olaparib inhibits PARP1 activity, thereby reducing PARylation levels, RIG-I lactylation further sensitizes tumors to olaparib (Li Y. et al., 2026). In colorectal cancer, pyruvate kinase M2 (PKM2) lactylation at K206 promotes its nuclear translocation and interaction with fos-related antigen 1 (FOSL1), ultimately driving vasculogenic mimicry and bevacizumab resistance (Li W. et al., 2026). In cellular senescence, early growth response 1 (EGR1) undergoes lactylation at K422, which promotes its nuclear translocation and subsequent *CDKN1A* transcription, ultimately inducing the senescence phenotype (Liang et al., 2026). Collectively, these examples illustrate that lactylation serves as a key signal driving the nuclear translocation of diverse proteins, thereby connecting metabolic states to nuclear functions.

## 3 Regulation of lactylation by metabolic enzymes

Lactylation is a metabolism-derived post-translational modification that depends on lactate as its substrate. Given that lactate is primarily produced through glycolysis, cellular lactylation levels are intimately linked to glucose metabolism. Indeed, fluctuations in glycolytic flux directly influence the availability of lactate, thereby shaping the global lactylation landscape. Consequently, glucose metabolism serves as a critical regulator of both histone and non-histone lactylation.

Metabolic enzymes play a central role in this regulatory axis by controlling the rate of lactate production. Enzymes such as phosphofructokinase (PFK), PKM2, and LDHA determine glycolytic output and, in turn, the intracellular concentration of lactate. As such, these enzymes function as key determinants of the lactylation landscape within cells. By modulating lactate availability, they indirectly govern the lactylation status of diverse protein substrates, bridging cellular metabolic state to post-translational regulation.

### 3.1 Metabolic enzymes regulate histone lactylation

Given their control over lactate production, several metabolic enzymes have been shown to influence histone lactylation, thereby regulating gene expression and disease progression. Here we highlight several representative examples. PFK promotes glycolysis and lactate production, which in turn enhances H3K18 lactylation. This epigenetic modification increases the transcriptional activity of *zinc finger E-box-binding homeobox 1* (*ZEB1*) and *contactin 1* (*CNTN1*), enhancing their protein levels and driving malignant phenotypes in tumor cells, including proliferation, migration, and invasion (Wang R. et al., 2024; Shen et al., 2025). Similarly, enolase 2 (ENO2) and triosephosphate isomerase 1 (TPI1) promote glycolysis and lactate accumulation, leading to H3K18 lactylation, which induces epithelial-mesenchymal transition (EMT) and subsequently promotes the development of myopia (Lin et al., 2024). LINC00183, a platelet-derived exosome component, binds to enolase 1 (ENO1) and enhances glycolysis, thereby driving H3K18 lactylation and promoting growth differentiation factor 15 (GDF15) expression. This axis ultimately supports the growth and metastasis of colorectal cancer (Su et al., 2025). Isocitrate dehydrogenase 3β (IDH3β), a tricarboxylic acid (TCA) cycle enzyme, provides an additional layer of regulation. In Alzheimer’s disease (AD) and AD-transgenic mice’s brains, reduced IDH3β expression uncouples OXPHOS, thereby enhancing glycolysis and leading to lactate accumulation. This metabolic shift drives lactylation of histone sites including H4K12 and H4K8, which in turn enhances the expression of paired-box gene 6 (PAX6). PAX6 then inhibits IDH3β expression, forming a positive feedback IDH3β-lactate-histone lactylation-PAX6-IDH3β loop that ultimately accelerates AD deterioration (Wang X. et al., 2024).

### 3.2 Metabolic enzymes regulate non-histone lactylation

In addition to regulating histone lactylation, glucose-metabolizing enzymes also promote lactylation of non-histone proteins, with broad functional implications. Chu et al. revealed that aldolase B (ALDOB) induces lactate production and activates pyruvate dehydrogenase kinase-1 (PDK1), thereby promoting lactylation of carcinoembryonic antigen cell adhesion molecule 6 (CEACAM6). This modification enhances CEACAM6 stability, leading to colon cancer cell proliferation and chemoresistance (Chu et al., 2023). Elevated glyceraldehyde-3-phosphate dehydrogenase (GAPDH) protein activity increases intracellular lactate concentration, which in turn promotes snail family transcriptional repressor 1 (SNAIL1) lactylation at K9, eventually resulting in fibroblast activation and pulmonary fibrosis (Wu J. et al., 2025). PDHA1, when inactivated through acetylation, increases intracellular lactate concentration via inhibition of the TCA cycle. This mediates lactylation of the mitochondrial fission 1 protein (FIS1) at K20, further promoting excessive mitochondrial fission and leading to mitochondrial apoptosis, ultimately resulting in sepsis-associated acute kidney injury (An et al., 2023). Phosphoenolpyruvate carboxykinase 1 (PCK1), a central gluconeogenic enzyme, binds to LDHA to promote lactylation of sterol regulatory element-binding protein (SREBP) regulating gene (SPRING) at K82. Enhanced mevalonate pathway activity resulting from this event inhibits ferroptosis and drives chemoresistance through the PCK1-LDHA-SPRINGlac axis (Zhu et al., 2025b).

## 4 Lactylation of metabolic enzymes

While metabolic enzymes regulate lactylation, accumulating evidence indicates that lactylation reciprocally modulates metabolic enzymes, highlighting a bidirectional interplay between metabolism and lactylation. Currently, two main mechanisms link these processes. The first involves lactylation of metabolic enzymes themselves, which directly alters their catalytic activity, stability, and molecular interactions (Figure 1). The second operates indirectly through lactylation of other proteins: histone lactylation modulates the expression of metabolic enzymes, while lactylation of other non-histone proteins also affects their expression or activity, thereby reprogramming cellular metabolism (Figure 1). Both mechanisms play critical roles in determining cell fate. This section focuses on the first: how lactylation of metabolic enzymes directly regulates their function and ultimately influences cell fate.

### 4.1 Proteomic analysis of the lactylation of metabolic enzymes

**FIGURE 3:**
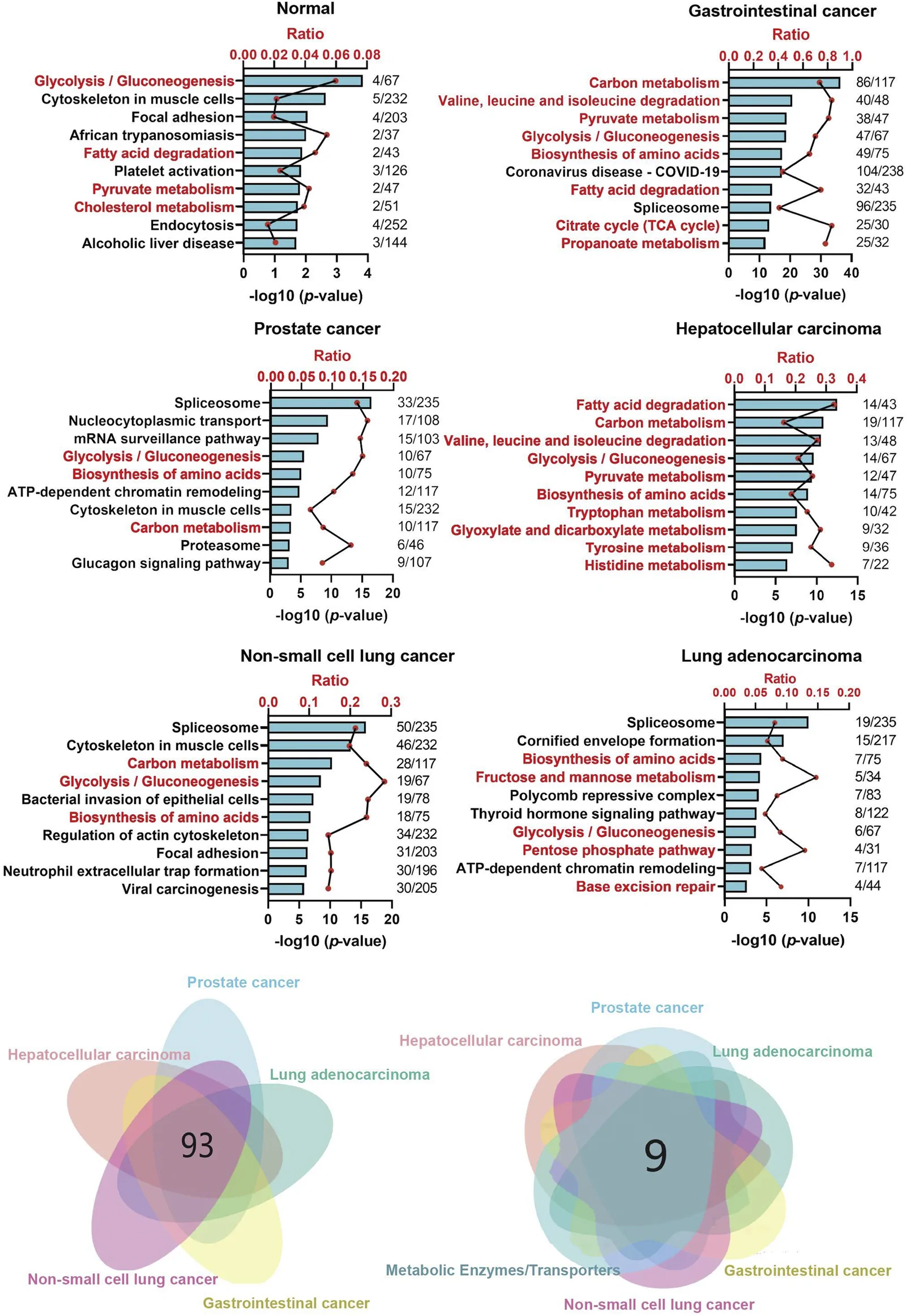
Metabolic enzymes are highly lactylated. Enrichment analyses of published lactylome datasets reveal that lactylation is widely distributed across metabolic pathways. These datasets cover various cell types including normal lung cells and several tumor cells such as those of lung adenocarcinoma, non-small cell lung cancer, hepatocellular carcinoma, prostate cancer, and gastrointestinal cancer. Subsequent integrative analysis of omics data from five tumor types identifies 93 proteins that are commonly lactylated in all five tumor types. Among these, nine are metabolic enzymes and transport proteins. All data on protein lactylation in normal cells and various cancer cell types, as well as information on metabolic enzymes and transport proteins, were obtained from the cited references. Pathway enrichment analysis: Normal (Yang Y. H. et al., 2023), Gastrointestinal cancer (Duan et al., 2024), Prostate cancer (Kim et al., 2025), Hepatocellular carcinoma (Hong et al., 2023), Non-small cell lung cancer (Chen et al., 2024b), Lung adenocarcinoma (Shan et al., 2025). Venn: Gastrointestinal cancer (Duan et al., 2024), Prostate cancer (Kim et al., 2025), Hepatocellular carcinoma (Hong H. et al., 2025), Non-small cell lung cancer (Chen et al., 2024b), Lung adenocarcinoma (He J. et al., 2025), Metabolic enzymes and transport proteins (Possemato et al., 2011). Created with Adobe Illustrator.

Comparative analysis of lactylation proteomics (lactylome) data from multiple studies has revealed a striking difference in lactylation levels between normal and malignant cells. Specifically, the number of lactylated proteins in normal cells is substantially lower than that in tumor cells (Yang Y. H. et al., 2023). Our pathway enrichment analysis of lactylated proteins, based on several published lactylome datasets, further showed that in both normal and tumor cells, metabolism-related pathways rank among the top ten enriched categories; in certain tumors, all ten of the most enriched pathways are metabolism-related. Moreover, these omics data demonstrated that proteins involved in carbon metabolism and the glycolysis/gluconeogenesis pathway are extensively lactylated (Figure 3). Collectively, these findings underscore the critical role of protein lactylation in metabolic pathways during tumor initiation and progression (Hong et al., 2023; Yang Y. H. et al., 2023; Chen et al., 2024b; Duan et al., 2024; Kim et al., 2025; Shan et al., 2025).

To gain deeper insight into the landscape of metabolic enzyme lactylation across cancer types (Figure 3), we analyzed five independent proteomics datasets derived from five distinct cancer cell types: hepatocellular carcinoma, prostate cancer, lung adenocarcinoma, gastrointestinal cancer, and non-small cell lung cancer (Chen et al., 2024b; Duan et al., 2024; He J. et al., 2025; Hong H. et al., 2025; Kim et al., 2025). The total numbers of lactylated proteins identified in these cell types were 960, 397, 1,218, 3,152, and 827, respectively. Among these, 93 proteins were commonly lactylated across all five tumor types. We further focused on metabolic enzymes and transporters, compiling a comprehensive list of 2,752 such proteins (Possemato et al., 2011). Among these, the numbers of lactylated metabolic enzymes and transporters identified in each of the five cancer types were 294, 19, 111, 551, and 111, respectively. Notably, nine metabolic enzymes and transporters, i.e., aldolase A (ALDOA), GAPDH, ENO1, glycogen phosphorylase L (PYGL), phosphofructokinase M (PFKM), adenosine kinase (ADK), phosphofructokinase P (PFKP), peroxiredoxin 1 (PRDX1), and ATP binding cassette subfamily F member 1 (ABCF1), were found to be lactylated in all five tumor types. Of these, ABCF1 is the sole transporter; the remaining eight are metabolic enzymes. Strikingly, six of these eight enzymes (ALDOA, GAPDH, ENO1, PFKM, PFKP, and PRDX1) are directly involved in glucose metabolism (Figure 3). This enrichment further reinforces the intimate relationship between glucose metabolism, global protein lactylation, and cancer progression.

### 4.2 Lactylation of enzymes in glucose metabolism

**FIGURE 4:**
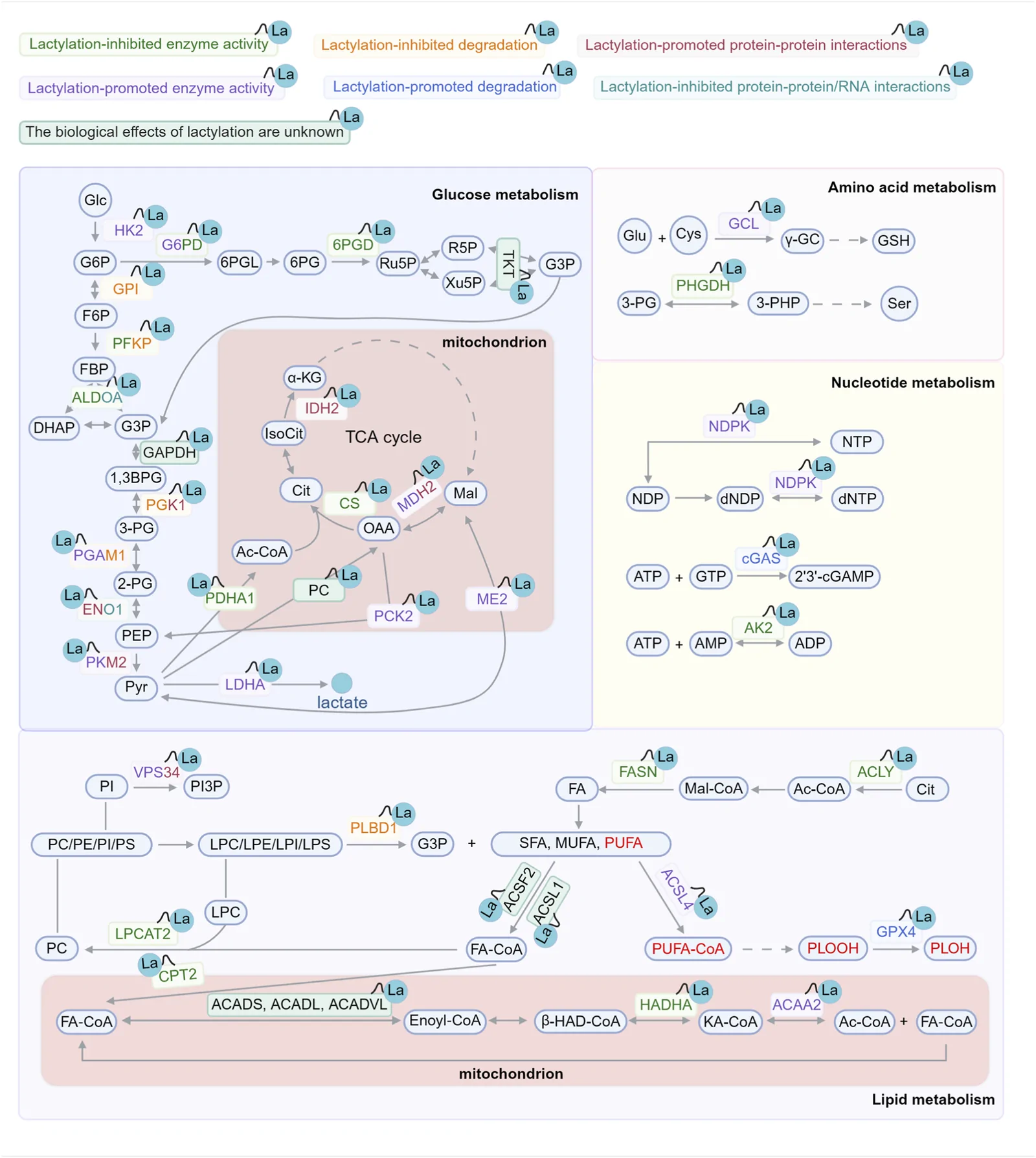
Lactylation of metabolic enzymes in four major metabolic pathways: glucose, lipid, amino acid, and nucleotide metabolism. Lactylation of metabolic enzymes occurs across multiple metabolic pathways. In glucose metabolism, lactylation affects enzymes involved in glycolysis, the TCA cycle, gluconeogenesis, and the PPP. In lipid metabolism, it targets enzymes in lipolysis, β-oxidation, lipid peroxidation, fatty acid synthesis, and membrane lipid synthesis. In amino acid metabolism, lactylation is found in glutathione metabolism and serine metabolism. In nucleotide metabolism, it impacts nucleotide synthesis and nucleotide interconversion. The effects of lactylation on enzyme activity, stability, and molecular interactions are indicated with distinct colors. If an enzyme is labeled with two colors, it indicates that lactylation at different sites leads to two distinct alterations. Black text indicates that lactylation has been confirmed to occur, but the resulting functional changes have not yet been studied. References for these enzymes: GAPDH (Wang C. et al., 2025), PC (Chen et al., 2023), TKT (Hong H. et al., 2025), ACSL1 (Wang F. et al., 2024), ACSF2 (Chen et al., 2024a), ACADS/ACADL/ACADVL (Hong H. et al., 2025; Li R. et al., 2025; Wang L. et al., 2025). Created with BioRender.com.

Previous studies have clearly shown that lactylation influences glucose metabolism and thereby affects multiple cellular processes. Glucose metabolism encompasses multiple pathways, including glycolysis, TCA cycle, gluconeogenesis, and pentose phosphate pathway (PPP), all of which are closely linked to protein lactylation (Figure 4; Table 1). In this section, we discuss how lactylation regulates key enzymes within each of these pathways.

**TABLE 1:** Lactylation sites and lactylation-induced functional changes of metabolic enzymes.

Metabolism	Metabolic enzymes	Function (lactylation site)	Disease	Reference
Glucose metabolism	HK2	Enhances enzyme activity (K346la)	Preeclampsia	Chen et al., 2025c
	GPI	Enhances stability (K454la)	Rheumatoid arthritis	Tan et al., 2025
	PFKP	Enhances stability (K392la) Inhibits enzyme activity (K688la)	Ovarian cancer Colorectal cancer	Mi et al., 2025; Cheng et al., 2024
	ALDOA	Inhibits enzyme activity (K147la) Inhibits protein-protein interaction (K230/322la)	Colorectal cancer hepatocellular carcinoma	Cheng et al., 2024; Feng et al., 2024
	GAPDH	Not studied (K263la)	Calcific aortic valve disease	Wang et al., 2025a
	PGK1	Enhances stability (K11la) Promotes protein-protein interaction (K361la)	Not studied Osseous injury	Wan et al., 2022; Qin et al., 2026
	PGAM1	Enhances enzyme activity (K251la) Enhances stability (K251la)	Hepatocellular carcinoma	Li et al., 2026b
	ENO1	Promotes protein-protein interaction (K89la) Inhibits protein-RNA interaction (K71la)	Lung adenocarcinoma Vascular injury	Gan et al., 2026; Xie et al., 2026
	PKM2	Enhances enzyme activity (K62la) Promotes protein-protein interaction (K206la)	Inflammation Colorectal cancer	Wang et al., 2022; Li et al., 2026c
	LDHA	Enhances enzyme activity (K81/318la)	Lung adenocarcinoma	Li et al., 2026a
	PDHA1	Inhibits enzyme activity (K336la)	Not studied	Mao et al., 2024
	CS	Inhibits enzyme activity (K370la)	Chronic kidney disease	Yu et al., 2025
	IDH2	Promotes protein-protein interaction (K272la)	Diabetic myocardial infarction	Zang et al., 2025
	MDH2	Enhances enzyme activity (K239la) Promotes protein-protein interaction (K239la)	Myocardial ischemia-reperfusion injury	Tang et al., 2025
		Not studied (K241la)	Renal cell carcinoma	She et al., 2024
	ME2	Enhances enzyme activity (K352la)	Colorectal cancer	Li et al., 2025a
	PC	Not studied	Not studied	Chen et al., 2023
	PCK2	Enhances enzyme activity (K100la)	Ischemia-reperfusion injury	Yuan et al., 2025
	G6PD	Enhances enzyme activity (K45-47la) Inhibits enzyme activity (K45la, K432la)	Lung adenocarcinoma Cervical cancer Ulcerative colitis	Zhang et al., 2025c; Meng et al., 2024b; Wu et al., 2026
	6PGD	Inhibits enzyme activity (K38la)	Ulcerative colitis	Wu et al., 2026
	TKT^*^	Not studied (K16la, K232la, K319la)	Not studied	Hong et al., 2025a
	UGDH	Inhibits enzyme activity (K6la)	Osteoarthritis	Lan et al., 2025
Lipid metabolism	PLBD1	Enhances stability (K155la)	Brain injury	Zhou et al., 2025a
	ACSL1^*^	Not studied (K676la)	Not studied	Wang et al., 2024a
	ACSF2	Not studied (K182la)	Diabetic kidney disease	Chen et al., 2024a
	ACSL4	Enhances enzyme activity (K412la)	Intervertebral disc degeneration	Sun et al., 2025b
	CPT2	Inhibits enzyme activity (K457/458la)	Not studied	Mao et al., 2024
	ACADS^*^ ACADL^*^ ACADVL^*^	Not studied (K133la) Not studied (K333la) Not studied (K298la)	Not studied	Hong et al., 2025a; Li et al., 2025d; Wang et al., 2025b
	HADHA	Inhibits enzyme activity (K166/728la)	Sepsis-induced cardiac dysfunction	Zhang et al., 2025b
	ACAA2	Enhances enzyme activity (K214la)	Breast cancer	Cui et al., 2025
	ACLY	Inhibits enzyme activity (K918/995la)	Not studied	Zhang et al., 2024c
	FASN	Inhibits enzyme activity (K673la)	Non-alcoholic fatty liver disease	Gao et al., 2023
	LPCAT2	Inhibits enzyme activity (K375la)	Sepsis-induced acute lung injury	Deng et al., 2026
	GPX4	Promotes degradation (K218/228la)	Myocardial ischemia-reperfusion injury	Wang et al., 2025d
	VPS34	Promotes protein-protein interaction (K356/781la)	Not studied	Jia et al., 2023
Amino acid metabolism	GCL	Enhances enzyme activity (K34la)	Pancreatic cancer	Chen et al., 2025b
	PHGDH	Inhibits enzyme activity (K21/69/289/394la)	Not studied	Trujillo et al., 2025
Nucleotide metabolism	NDPK	Enhances enzyme activity (K49la)	Glioblastoma	Batsios et al., 2026
	cGAS	Promotes degradation (K21la)	Lung adenocarcinoma	Rao et al., 2025
	AK2	Inhibits enzyme activity (K28la)	Hepatocellular carcinoma	Yang et al., 2023b

Enzymes marked with “*” are lactylated metabolic enzymes that were identified in published proteomics data and have not been extensively studied.

#### 4.2.1 Glycolysis

Extensive studies have shown that several glycolytic enzymes undergo lactylation, with functional outcomes that are often site- and context-dependent (Wan et al., 2022). Lactylation of hexokinase 2 (HK2) at K346 enhances its enzymatic activity and promotes its binding to voltage-dependent anion channel 1 (VDAC1), potentially contributing to enhanced glycolytic capacity and cell proliferation (Chen Z. et al., 2025). Lactylation of glucose-6-phosphate isomerase (GPI) at K454 enhances its stability, which may contribute to the proliferation, migration, and inflammatory responses of fibroblast-like synoviocytes and exacerbate rheumatoid arthritis (Tan et al., 2025). As a key rate-limiting enzyme in glycolysis, PFKP is lactylated at two distinct sites with opposing effects. Lactylation at K392 inhibits PFKP degradation and suppresses phosphatase and tensin homolog (PTEN) expression, thereby promoting glycolysis in ovarian cancer cells and accelerating tumor progression (Mi et al., 2025). In contrast, lactylation at K688 directly suppresses PFKP enzymatic activity, potentially contributing to the inhibition of colorectal cancer progression (Cheng et al., 2024). ALDOA was initially reported to undergo lactylation at K147, which may reduce its enzymatic activity, though its functional significance remains unclear. Subsequent studies identified lactylation at K230 and K322, which inhibits ALDOA binding to DDX17, thereby potentially contributing to maintenance of tumor stemness and the progression of hepatocellular carcinoma (Cheng et al., 2024; Feng et al., 2024). Beyond lactylation, GAPDH exemplifies how different acylations can compete for the same residue. GAPDH is lactylated at K263, but this site can also be butyrylated by gut microbiota-derived butyric acid. When the level of lactylation at the GAPDH K263 site exceeds that of butyrylation, it may contribute to the onset and progression of calcific aortic valve disease (Wang C. et al., 2025). The effects of lactylation on phosphoglycerate kinase 1 (PGK1) are cell-type specific. In A549 cells, lactylation at K11 enhances its thermal stability, an effect not observed in Jurkat cells (Wan et al., 2022). Additionally, lactylation at K361 regulates voltage-dependent anion channel 3 (VDAC3) and triggers FtMt/PINK1/Parkin-mediated mitophagy, thereby inducing ferroptosis in osteoblasts (Qin et al., 2026). Phosphoglycerate mutase 1 (PGAM1) undergoes competitive lactylation and ubiquitination at K251. JOSD1 removes K251-linked ubiquitination and prevents ubiquitin–proteasome-mediated degradation of PGAM1, thereby promoting lactylation at this site and enhancing its enzymatic activity, ultimately driving the malignant progression of hepatocellular carcinoma (Li Q. et al., 2026). ENO1 undergoes lactylation at two distinct sites with divergent functional outcomes. Lactylation at K89 promotes ENO1 interactions with pyruvate kinase M1 (PKM1), PKM2, lactate dehydrogenase B (LDHB), and malate dehydrogenase 2 (MDH2), driving metabolic reprogramming and conferring resistance to osimertinib in lung adenocarcinoma (Gan et al., 2026). In contrast, lactylation at K71 reduces *TRIM21* mRNA binding to ENO1 by limiting CCR4-NOT transcription complex subunit 6 (CNOT6) recruitment, thereby stabilizing *TRIM21* mRNA. Elevated TRIM21 promotes vascular endothelial cadherin (VE-cadherin) ubiquitination and degradation, disrupting endothelial adherens junctions and increasing permeability. Notably, a specific peptide targeting ENO1 K71 lactylation alleviated endothelial injury and improved survival in septic mice (Xie et al., 2026). PKM2 is lactylated at multiple sites with divergent outcomes. Lactylation at K62 maintains its tetrameric state, enhancing enzymatic activity and reducing nuclear translocation, thereby suppressing inflammatory responses (Wang et al., 2022). In contrast, lactylation at K206 promotes its nuclear translocation and interaction with FOSL1, facilitating FOSL1-dependent super-enhancer formation and target gene transcription, thereby driving vascular mimicry in colorectal cancer, enabling tumor cells to establish an alternative blood supply independent of canonical vascular endothelial growth factor A (VEGF-A)-mediated angiogenesis, thereby reducing their sensitivity to bevacizumab, a monoclonal antibody targeting VEGF-A (Li W. et al., 2026). LDHA is lactylated at K81 and K318. This modification enhances its enzymatic activity and, through a positive feedback loop, increases global intracellular lactylation levels, ultimately promoting cisplatin resistance in cancer cells (Li J. et al., 2026). Collectively, these findings demonstrate that lactylation of glycolytic enzymes is a widespread regulatory mechanism that fine-tunes enzyme activity, stability, and protein-protein interactions, thereby influencing a broad spectrum of diseases.

#### 4.2.2 TCA cycle

As the central hub of glucose oxidation, the TCA cycle is critical for both energy production and biosynthesis. Notably, a growing number of studies have reported that several key enzymes within this cycle—including PDHA1, citrate synthase (CS), isocitrate dehydrogenase 2 (IDH2), MDH2, and ME2—undergo lactylation, underscoring the intimate crosstalk between this modification and mitochondrial metabolism. PDHA1, which bridges glycolysis and the TCA cycle, is lactylated by AARS2 at K336. This modification reduces PDHA1 activity, impairing pyruvate utilization in mitochondria, limiting OXPHOS, and shifting cellular energy production toward glycolysis (Mao et al., 2024). In a mouse model of the transition from acute kidney injury to chronic kidney disease, CS lactylation at K370 was shown to impair its enzymatic activity, leading to mitochondrial dysfunction and NLRP3 inflammasome activation, thereby promoting renal injury. However, the K370 residue is not conserved in human CS, and whether this lactylation-mediated mechanism contributes to kidney injury in humans remains to be determined (Yu et al., 2025). Within the IDH family, IDH2—rather than IDH1 and IDH3—has been shown to undergo lactylation. Lactylation of IDH2 at K272 does not affect glucose metabolism but instead facilitates its binding to caveolin-1, while inhibiting the interaction between caveolin-1 and endothelial nitric oxide synthase (eNOS). This promotes eNOS phosphorylation and activity, stimulating cardiac microvascular endothelial cell proliferation and migration (Zang et al., 2025). MDH2 has two functionally distinct lactylation sites. Lactylation at K239, written by KAT8 and erased by SIRT3, enhances MDH2 activity and strengthens its interaction with the citrate transporter solute carrier family 25 member 1 (SLC25A1), thereby reprogramming mitochondrial metabolism, enhancing the antioxidant stress response, and promoting renal cell carcinoma progression (Tang et al., 2025). In contrast, lactylation at K241 of MDH2 induces ferroptosis by impairing mitochondrial function, potentially leading to myocardial ischemia-reperfusion injury (She et al., 2024). ME2, which links glucose metabolism, NADPH production, and lipid metabolism, undergoes lactylation at K352. This modification enhances its enzymatic activity and promotes mitochondrial function. Conversely, SIRT3-mediated delactylation at the same site disrupts redox homeostasis and inhibits tumor growth (Li C. et al., 2025).

#### 4.2.3 Gluconeogenesis

Only four enzymes in gluconeogenesis are not shared with glycolysis. Among these, pyruvate carboxylase (PC) has been confirmed to undergo lactylation, though the specific sites and functions remain unidentified (Chen et al., 2023). Phosphoenolpyruvate carboxykinase 2 (PCK2) is a key rate-limiting enzyme in gluconeogenesis that catalyzes the conversion of mitochondrial oxaloacetate to phosphoenolpyruvate, thereby linking the TCA cycle to glycolysis and gluconeogenesis. Lactylation of PCK2 at K100 competitively inhibits Parkin-mediated polyubiquitination of 3-oxoacyl-ACP synthase, mitochondrial (OXSM), remodeling mitochondrial fatty acid synthesis, potentiating OXPHOS and the TCA cycle, and ultimately inducing ferroptosis during ischemia-reperfusion injury (Yuan et al., 2025). In contrast, PCK1, fructose-1,6-bisphosphatase, and glucose-6-phosphatase have no reported lactylation sites to date.

#### 4.2.4 Pentose phosphate pathway

The PPP branches from the first committed step of glycolysis. Instead of generating ATP, it primarily produces NADPH for biosynthesis and ribose-5-phosphate for nucleotide synthesis. Glucose-6-phosphate dehydrogenase (G6PD), a rate-limiting enzyme in the PPP, is subject to complex, context-dependent regulation by lactylation. In A549 cells, lactylation at K45-47 enhances G6PD’s NADP^+^ binding and dimerization, increasing its activity and promoting cell proliferation and migration (Zhang Y. et al., 2025). In contrast, in C33A cervical cancer cells expressing human papillomavirus type 16 early protein 6 (HPV16 E6), lactylation at the same K45 site produces an opposite effect: inhibition of lactylation promotes G6PD dimerization and activity, thereby activating the PPP and driving cell proliferation (Meng et al., 2024b). Additionally, lactylation of G6PD at K432 and 6-phosphogluconate dehydrogenase (6PGD) at K38 inhibits G6PD homodimer formation and 6PGD-NADP^+^ binding, suppressing their activity and potentially disrupting the intestinal mucosal barrier (Wu et al., 2026). Transketolase (TKT) has been reported to undergo lactylation, potentially promoting tumor development and atrial fibrillation (Jiao et al., 2024; Ma et al., 2025). Although direct experimental validation of specific sites is still lacking, omics data identified potential lactylation sites at K16, K232, and K319 (Hong H. et al., 2025). Further experimental validation is required to confirm these sites.

#### 4.2.5 Other glucose metabolism-related enzymes

Glycosylation is a fundamental branch of glucose metabolism that regulates protein function, cell signaling, and extracellular matrix composition. A key aspect of glycosylation is the synthesis of glycosaminoglycans, which are essential for maintaining tissue homeostasis and modulating inflammatory responses (Taylor and Gallo, 2006). UDP-glucose dehydrogenase (UGDH) is a critical enzyme in this pathway, catalyzing the conversion of UDP-glucose to UDP-glucuronic acid. A recent study has shown that p300-mediated lactylation of UGDH at K6 inhibits its enzymatic activity, reduces glycosaminoglycan synthesis, and disrupts signal transducer and activator of transcription 1 (STAT1) interaction, thereby promoting *mitogen-activated protein kinase kinase kinase 8* (*MAP3K8*) transcription and activating the MAPK signaling pathway (Lan et al., 2025). Notably, the p300 inhibitor A485 suppresses UGDH lactylation and alleviates chondrocyte extracellular matrix degradation and osteoarthritis progression *in vitro* and *in vivo* (Lan et al., 2025).

### 4.3 Lactylation of enzymes in lipid metabolism

Lipid metabolism is essential for energy homeostasis, membrane biosynthesis, and signal transduction (Paulo et al., 2026). Lactylation of lipid metabolic enzymes has emerged as a key regulatory mechanism that influences diverse biological functions, spanning both catabolic and anabolic processes (Figure 4; Table 1).

#### 4.3.1 Lactylation of enzymes in lipid catabolism

Lipid catabolism is primarily an energy-producing process encompassing lipolysis, fatty acid β-oxidation, and ketogenesis (Paulo et al., 2026). To date, direct evidence supporting the lactylation of lipolytic enzymes remains lacking. Nevertheless, prolonged lactate exposure and high-intensity interval training have been shown to influence neural plasticity, accompanied by both elevated pan-lactylation and altered expression of hormone-sensitive lipase (HSL), which hydrolyzes diacylglycerols and cholesteryl esters. These observations raise the possibility that HSL may undergo lactylation or that histone lactylation may promote HSL expression, though the underlying mechanism requires further validation (Lei et al., 2024). In the context of glycerophospholipid catabolism, phospholipase B domain-containing protein 1 (PLBD1), a widely conserved enzyme responsible for removing fatty acids from glycerophospholipids, has been shown to undergo lactylation at K155. This modification enhances PLBD1 stability and increases its protein levels, ultimately exacerbating brain damage following ischemic stroke (Zhou F. et al., 2025).

Acyl-CoA synthetase (ACS) family members catalyze the “activation” of free fatty acids by attaching them to CoA to form fatty acyl-CoA thioesters. This crucial process allows intracellular fatty acids to enter complex lipid biosynthesis, membrane remodeling, and energy production pathways like β-oxidation. Among this family, acyl-CoA synthetase long-chain family member 1 (ACSL1) has been shown to undergo lactylation at K676, although the underlying mechanism remains unclear (Wang F. et al., 2024). Other ACS members exhibit lactylation with distinct functional outcomes. Specifically, ACSL4 lactylation at K412 enhances its activity and promotes ferroptosis (Sun K. et al., 2025), whereas acyl-CoA synthetase family member 2 (ACSF2) lactylation at K182 inhibits mitochondrial function, contributing to the progression of diabetic kidney disease (DKD) (Chen et al., 2024a).

Following acyl-CoA synthesis, CPT2 transports long-chain acyl-CoA into mitochondria. Lactylation of CPT2 at K457/458 reduces or abolishes its enzymatic activity, thereby inhibiting fatty acid β-oxidation and suppressing OXPHOS (Mao et al., 2024). The acyl-CoA dehydrogenases ACADS, ACADL, and ACADVL catalyze the dehydrogenation of short-chain, long-chain, and very long-chain fatty acids, respectively. Although all three undergo lactylation, no functional studies have been reported to date. Omics data have identified potential lactylation sites at K133 (ACADS), K333 (ACADL), and K298 (ACADVL) (Zhang T. et al., 2024; Hong H. et al., 2025; Li R. et al., 2025; Wang L. et al., 2025). Hydroxyacyl-CoA dehydrogenase trifunctional multienzyme complex subunit alpha (HADHA), a key rate-limiting enzyme in β-oxidation, undergoes lactylation at K166 and K728. This modification inhibits its activity, disrupts fatty acid metabolism, impairs mitochondrial function and ATP production, thereby contributing to sepsis-induced cardiac dysfunction (Zhang T. N. et al., 2025). Acetyl-CoA acyltransferase 2 (ACAA2) catalyzes the terminal and rate-limiting step of mitochondrial fatty acid β-oxidation. A recent study revealed that lactate dehydrogenase C4 (LDHC4) promotes lactylation of ACAA2 at K214, which enhances its catalytic activity and accelerates fatty acid metabolism. This increased metabolic flux leads to elevated production and accumulation of free fatty acids, subsequently inducing autophagy and contributing to the progression of triple-negative breast cancer (Cui et al., 2025). It is worth noting that the lactylated metabolic enzymes involved in the β-oxidation process discussed in this section and those involved in the TCA cycle described in Section 4.2.2 are localized within mitochondria. Based on their subcellular localization, these proteins can be categorized as mitochondrial metabolic enzymes, which have been shown to undergo extensive lactylation. Recent studies have demonstrated that lactate can enter mitochondria and contribute to AARS2-mediated lactylation modification within this organelle (Brooks, 2018; Mao et al., 2024). Therefore, research focusing on lactylation modifications of mitochondrial enzyme proteins may represent an important direction for future studies.

#### 4.3.2 Lactylation of enzymes in lipid anabolism

Lipid anabolism is essential for cellular survival, encompassing the biosynthesis of fatty acids, membrane phospholipids, and other structural lipids that constitute cellular membranes. Emerging evidence indicates that several key anabolic enzymes are subject to lactylation, which differentially modulates their activities and downstream physiological outcomes. ATP-citrate lyase (ACLY) generates acetyl-CoA, the fundamental building block for *de novo* fatty acid synthesis. ACLY exists as two splice variants—Acly Long (Acly L) and Acly Short (Acly S)—whose production is regulated by RNA-binding motif protein 25 (RBM25). Notably, only Acly L undergoes lactylation at K918/995, which inhibits its enzymatic activity and disrupts substrate binding, thereby suppressing metabolic activity and inflammatory responses (Zhang Y. et al., 2024). Downstream of ACLY, fatty acid synthase (FASN) is a multifunctional complex that catalyzes the condensation, reduction, dehydration, and re-reduction reactions required for fatty acid chain elongation. Lactylation of FASN at K673 inhibits its synthase activity, consequently disrupting hepatic fatty acid biosynthesis (Gao et al., 2023).

The metabolic enzyme lysophosphatidylcholine acyltransferase 2 (LPCAT2) participates in membrane lipid remodeling, a key aspect of phospholipid anabolism. LPCAT2 undergoes lactylation at K375, which impairs its acetyltransferase activity. This leads to increased STAT1 phosphorylation and subsequent transcriptional repression of *SLC7A11*, ultimately inducing ferroptosis in epithelial cells and contributing to the progression of sepsis-induced acute lung injury (Deng et al., 2026). Glutathione peroxidase 4 (GPX4) protects membrane lipids from oxidative damage, thereby inhibiting ferroptosis and preserving membrane integrity. A recent study demonstrated that 2-DG suppresses lactylation of GPX4 at K218 and K228, while simultaneously increasing its protein stability. Notably, GPX4 overexpression alleviated injury induced by hypoxia/reoxygenation (H/R) and attenuated cardiomyocyte ferroptosis (Wang Y. et al., 2025). Vacuolar protein sorting 34 (VPS34) exhibits lipokinase activity, generating phosphatidylinositol 3-phosphate (PI3P) to initiate autophagic membrane formation, a process intimately linked to membrane lipid turnover. Lactylation of VPS34 at K356 and K781 by KAT5/TIP60 enhances its binding to Beclin1, autophagy-related 14-like protein (ATG14L), and UV radiation resistance-associated gene (UVRAG), thereby promoting VPS34 lipokinase activity and subsequently contributing to autophagosome formation and maturation (Jia et al., 2023).

For other branches of lipid anabolism, including cholesterol anabolism, lipid droplet formation, and phospholipid anabolism beyond LPCAT2-mediated remodeling, no studies to date have demonstrated lactylation of the relevant metabolic enzymes.

### 4.4 Lactylation of enzymes in amino acid metabolism

Amino acids serve as the fundamental building blocks of proteins, which execute virtually all biological processes. To date, research on the lactylation of enzymes involved in amino acid metabolism remains relatively limited (Figure 4; Table 1). In glutathione metabolism, the regulatory subunit of glutamate-cysteine ligase (GCL), known as glutamate-cysteine ligase M (GCLM), undergoes lactylation at K34. This modification enhances GCL enzymatic activity, leading to increased GSH synthesis and conferring resistance to ferroptosis in G12D mutant cancer cells (Chen Y. et al., 2025). In the serine metabolic pathway, phosphoglycerate dehydrogenase (PHGDH), an enzyme involved in serine synthesis, contains multiple lactylation sites at K21, K69, K289, and K394. Notably, lactylation of PHGDH occurs potentially through the formation of K_D-la_, in which LGSH derived from the glyoxalase pathway serves as the lactyl donor. This modification may inhibit PHGDH activity, thereby potentially impairing the conversion of 3-phosphoglycerate to serine and reducing intracellular serine synthesis (Trujillo et al., 2025).

### 4.5 Lactylation of enzymes in nucleotide metabolism

In nucleotide synthesis (Figure 4; Table 1), nucleoside diphosphate kinase (NDPK) undergoes lactylation at K49, which enhances its activity, promotes the conversion of NDP to NTP, and facilitates the production of precursors for nucleotide and deoxyribonucleotide synthesis (Batsios et al., 2026). Separately, cGAS functions as a cytosolic DNA sensor that catalyzes the sequential condensation of ATP and GTP to form 2′,3′-cyclic GMP-AMP, thereby activating innate immune responses. However, accumulated lactate induces lactylation of cGAS at K21, which facilitates its translocation to the proteasome for degradation, thus attenuating its immune function (Rao et al., 2025). Adenylate kinase 2 (AK2) is a mitochondrial enzyme that regulates adenine nucleotide interconversion within the intermembrane space. Lactylation of AK2 at K28 inhibits its function, thereby potentially facilitating the proliferation and metastasis of hepatocellular carcinoma cells (Yang Z. et al., 2023).

## 5 The effects of lactylation on metabolic enzymes: catalysis, turnover, and molecular networks

**FIGURE 5:**
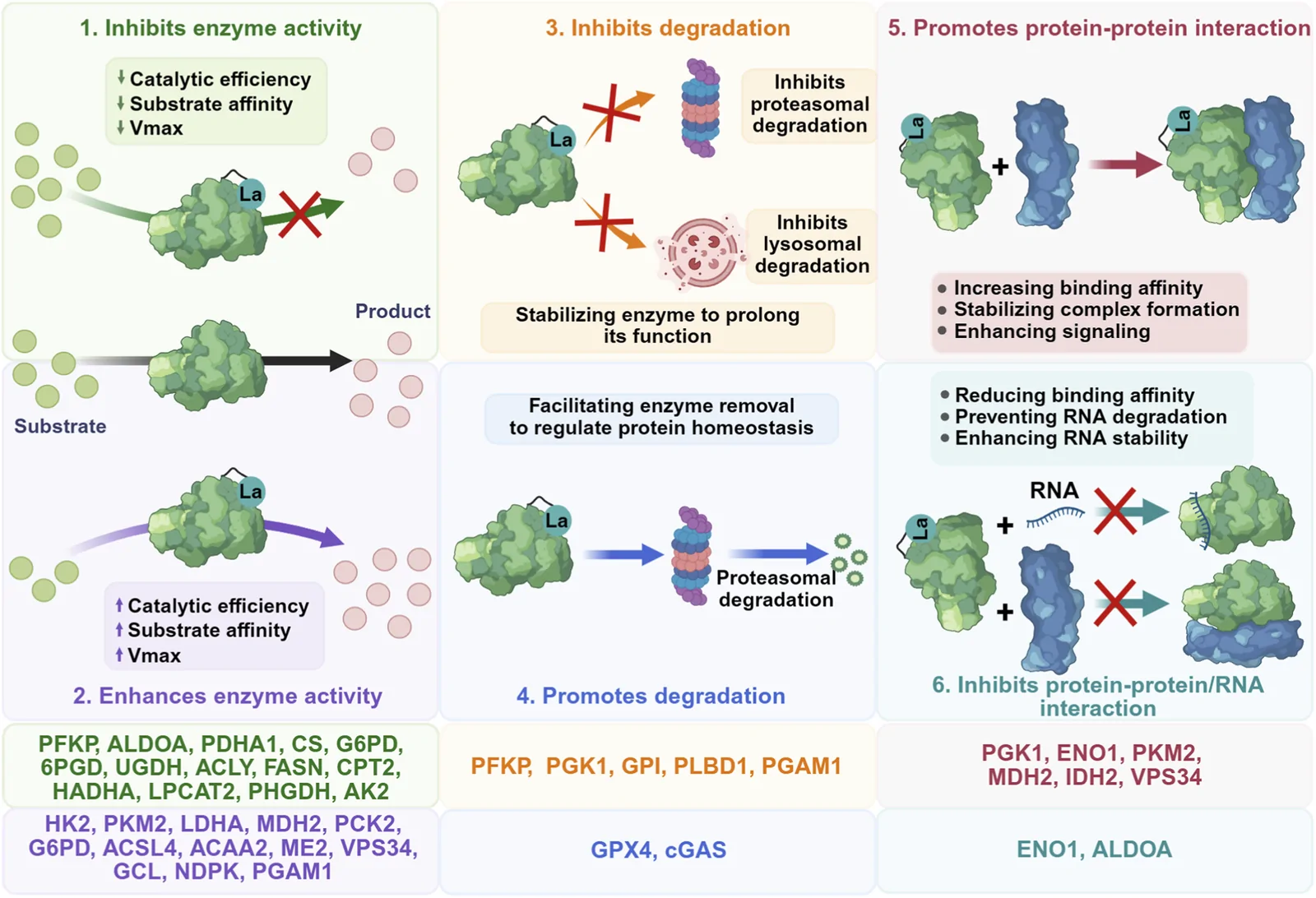
Lactylation affects the properties of metabolic enzymes. Lactylation of metabolic enzymes alters their functional properties. These changes manifest in three ways: modulation of enzyme activity either enhancing or reducing it, alteration of protein degradation, and regulation of interactions between proteins and other molecules. Created with BioRender.com.

Given that comprehensive reviews have extensively summarized the epigenetic regulation of metabolic enzyme expression and its pathophysiological significance, this paper will not elaborate further on these topics. Instead, we focus on how lactylation modification alters the intrinsic properties of metabolic enzymes. Although Section 3 discusses lactylation of individual metabolic enzymes in detail, this section revisits these findings and provides a brief classification and summary of the properties of metabolic enzymes that are altered by this modification. Specifically, lactylation modulates catalytic activity, influences protein degradation, and mediates enzyme-molecule interactions (Figure 5; Table 1).

### 5.1 Lactylation modulates metabolic enzyme activity

Emerging evidence indicates that lactylation exerts a bidirectional regulatory effect on metabolic enzyme activity, functioning as either an inhibitor or an activator depending on the specific enzyme and the cellular context.

#### 5.1.1 Lactylation suppresses enzyme catalytic activity

For a range of metabolic enzymes, lactylation serves as an inhibitory modification.

In glucose metabolism, lactylation at K688 of PFKP inhibits its activity, potentially suppressing colorectal cancer progression (Cheng et al., 2024). ALDOA undergoes lactylation at K147, a residue critical for substrate binding, resulting in diminished enzymatic function (Wan et al., 2022; Cheng et al., 2024). Lactylation of PDHA1 at K336 by AARS2 inhibits its activity, which in turn shifts the metabolic balance toward glycolysis (Mao et al., 2024). In the TCA cycle, lactylation of CS at K370 inhibits its enzymatic activity, ultimately leading to kidney injury (Yu et al., 2025). G6PD and 6PGD undergo lactylation at K432 and K38, respectively, with both modifications inhibiting their enzymatic activities and thereby potentially protecting the intestinal mucosal barrier (Wu et al., 2026). Furthermore, lactylation of UGDH at K6 reduces its enzymatic activity, and ultimately exacerbates osteoarthritis (Lan et al., 2025).

In lipid metabolism, lactylation similarly exerts inhibitory effects. ACLY activity is inhibited by lactylation at K918/K995, compromising metabolic activity and attenuating inflammatory responses (Zhang Y. et al., 2024). FASN is subject to lactylation at K673, which inhibits fatty acid synthesis (Gao et al., 2023). CPT2 undergoes lactylation at K457/K458, which inhibits its enzymatic activity, thereby suppressing OXPHOS (Zhou F. et al., 2025). Lactylation of HADHA at K166/K728 impairs its enzymatic activity, thereby contributing to sepsis-induced cardiac dysfunction (Zhang T. N. et al., 2025). In membrane lipid metabolism, lactylation of LPCAT2 at K375 reduces its acetyltransferase enzymatic activity, inducing ferroptosis and contributing to the progression of sepsis-induced acute lung injury (Deng et al., 2026).

In amino acid metabolism, the serine-metabolizing enzyme PHGDH undergoes lactylation at multiple sites (K21, K69, K289, and K394). This modification potentially inhibits its enzymatic activity and potentially impairs intracellular serine synthesis (Trujillo et al., 2025).

Within nucleotide homeostasis, lactylation of AK2 at K28 impairs its enzymatic function, thereby potentially promoting the proliferation and metastasis of hepatocellular carcinoma cells (Yang Z. et al., 2023).

#### 5.1.2 Lactylation potentiates enzyme catalytic activity

In contrast to the inhibitory effects described above, lactylation can also enhance the catalytic activity of several metabolic enzymes. In glucose metabolism, lactylation at K346 of HK2 enhances its enzymatic activity, contributing to the pathogenesis of preeclampsia (PE) (Chen Z. et al., 2025). PGAM1 undergoes lactylation at K251, which enhances its enzymatic activity, and ultimately promotes the progression of hepatocellular carcinoma (Li Q. et al., 2026). PKM2 undergoes lactylation at K62, which enhances its enzymatic activity and suppresses inflammatory responses (Wang et al., 2022). LDHA is activated by lactylation at K81 and K318, leading to increased intracellular lactate levels and resistance to cisplatin (Li J. et al., 2026). MDH2 exhibits enhanced enzymatic activity following K239 lactylation, which remodels mitochondrial metabolism and accelerates renal cancer progression (Tang et al., 2025). PCK2 shows increased activity after K100 lactylation, ultimately leading to liver injury (Yuan et al., 2025). The effect of lactylation on G6PD is context-dependent. Lactylation at K45-47 enhances G6PD activity. In C33A cells infected with HPV16, however, G6PD activity is increased only when K45 lactylation is blocked, leading to promotion of the malignant phenotypes in tumor cells (Meng et al., 2024b; Zhang Y. et al., 2025).

In lipid metabolism, lactylation at K412 of ACSL4 enhances its enzymatic activity, thereby promoting ferroptosis (Sun K. et al., 2025). ACAA2 exhibits enhanced catalytic activity following K214 lactylation, contributing to triple-negative breast cancer progression (Cui et al., 2025). ME2, which supplies NADPH for lipid metabolism, shows increased catalytic activity after K352 lactylation, promoting tumor progression (Li C. et al., 2025). VPS34 lactylation at K356 and K781 enhances its binding to Beclin1, ATG14L, and UVRAG, promoting its lipokinase activity and autophagosome formation/maturation (Jia et al., 2023).

In amino acid metabolism, specifically the glutathione metabolic pathway, GCLM enhances GCL activity through its lactylation at K34, inducing resistance to ferroptosis in KRAS G12D mutant cancer cells (Chen Y. et al., 2025).

Finally, in nucleotide metabolism, NDPK undergoes lactylation at K49, which enhances its enzymatic activity and promotes the synthesis of nucleotides and deoxyribonucleotides (Batsios et al., 2026).

### 5.2 Lactylation modulates the stability of metabolic enzymes

Beyond modulating catalytic activity, lactylation also affects the degradation of metabolic enzymes, thereby influencing their stability. Notably, the same enzyme can exhibit distinct functional outcomes depending on which residue is lactylated. In glucose metabolism, PFKP provides a clear example of this site-specific effect. As discussed in Section 5.1.1, lactylation at K688 of PFKP inhibits its enzymatic activity (Cheng et al., 2024). However, when lactylation occurs at a different residue, K392, it produces an opposing effect. Lactylation at K392 inhibits PFKP degradation, thereby promoting ovarian cancer progression (Mi et al., 2025). Similarly, lactylation at K11 of PGK1 in A549 cells enhances its protein stability and protects it from degradation (Wan et al., 2022). Interestingly, lactylation at a single residue can exert dual regulatory effects by simultaneously enhancing enzymatic activity and protein stability. PGAM1 undergoes competitive lactylation and ubiquitination at K251. Lactylation at this site suppresses ubiquitination, thereby preventing ubiquitin–proteasome-mediated degradation and enhancing protein stability (Li Q. et al., 2026). Notably, lactylation of certain metabolic enzymes regulates protein stability alone, without altering their enzymatic activity. Lactylation of GPI at K454 also enhances its stability, potentially exacerbating rheumatoid arthritis (Tan et al., 2025). Within glycerophospholipid catabolism, PLBD1 undergoes lactylation at K155. This post-translational modification enhances PLBD1 stability, thereby contributing to exacerbated brain damage following ischemic stroke (Zhou F. et al., 2025).

Of note, although lactylation appears to predominantly enhance metabolic enzyme stability by inhibiting ubiquitination, this is not a universal rule. A recent study has demonstrated that the lactylation of GPX4 at K218 and K228 reduces its protein stability, ultimately promoting cardiomyocyte ferroptosis (Wang Y. et al., 2025). Lactylation of cGAS at K21, induced by accumulated lactate, facilitates its translocation to the proteasome for degradation, representing a distinct mechanism that promotes protein decay (Rao et al., 2025).

### 5.3 Lactylation modulates the binding of metabolic enzymes to other molecules

Beyond altering enzyme activity and stability, lactylation can also influence the capacity of metabolic enzymes to interact with other biomolecules, including proteins and RNA. Emerging evidence indicates that such modifications can either promote or disrupt these interactions, thereby exerting diverse functional consequences.

Several studies have demonstrated that lactylation affects protein-protein interactions involving metabolic enzymes. In glucose metabolism, lactylation of ALDOA at K230 and K322 inhibits its binding to DDX17, thereby potentially maintaining tumor stemness (Feng et al., 2024). Lactylation of PGK1 at K361 regulates its interaction with VDAC3, potentially contributing to mitochondrial damage and ferroptosis (Qin et al., 2026). ENO1 undergoes lactylation at K89, which enhances its interaction with metabolic enzymes such as PKM1, PKM2, and LDHB, and eventually accelerating resistance to osimertinib (Gan et al., 2026). PKM2 is subject to lactylation at K206, which promotes its nuclear translocation and interaction with FOSL1, ultimately facilitating vascular mimicry in colorectal cancer and conferring resistance to bevacizumab (Li W. et al., 2026). Lactylation of MDH2 at K239 strengthens the interaction between MDH2 and the citrate transporter SLC25A1, ultimately promoting renal cell carcinoma progression (Tang et al., 2025). IDH2, when lactylated at K272, facilitates its binding to caveolin-1, eventually improving cardiac repair following diabetic myocardial infarction (Zang et al., 2025). Lactylation of VPS34 at K356 and K781 enhances its binding to Beclin1, ATG14L, and UVRAG, thereby contributing to autophagosome formation and maturation (Jia et al., 2023).

Xie et al. reported that elevated lactate levels during sepsis promote lactylation of ENO1 at K71, an event facilitated by increased activity of the lactyltransferase p300. This modification reduces the binding of ENO1 to *TRIM21* mRNA, thereby preventing its degradation by limiting the recruitment of CNOT6 (Xie et al., 2026). Together, these examples illustrate that lactylation not only influences the interplay between catalytic and RNA-binding functions of metabolic enzymes but also expands their regulatory repertoire beyond classical control, enabling dynamic interactions with diverse molecular partners.

## 6 Pathological implications of metabolic enzyme lactylation

### 6.1 Lactylation of metabolic enzymes in tumor progression

**FIGURE 6:**
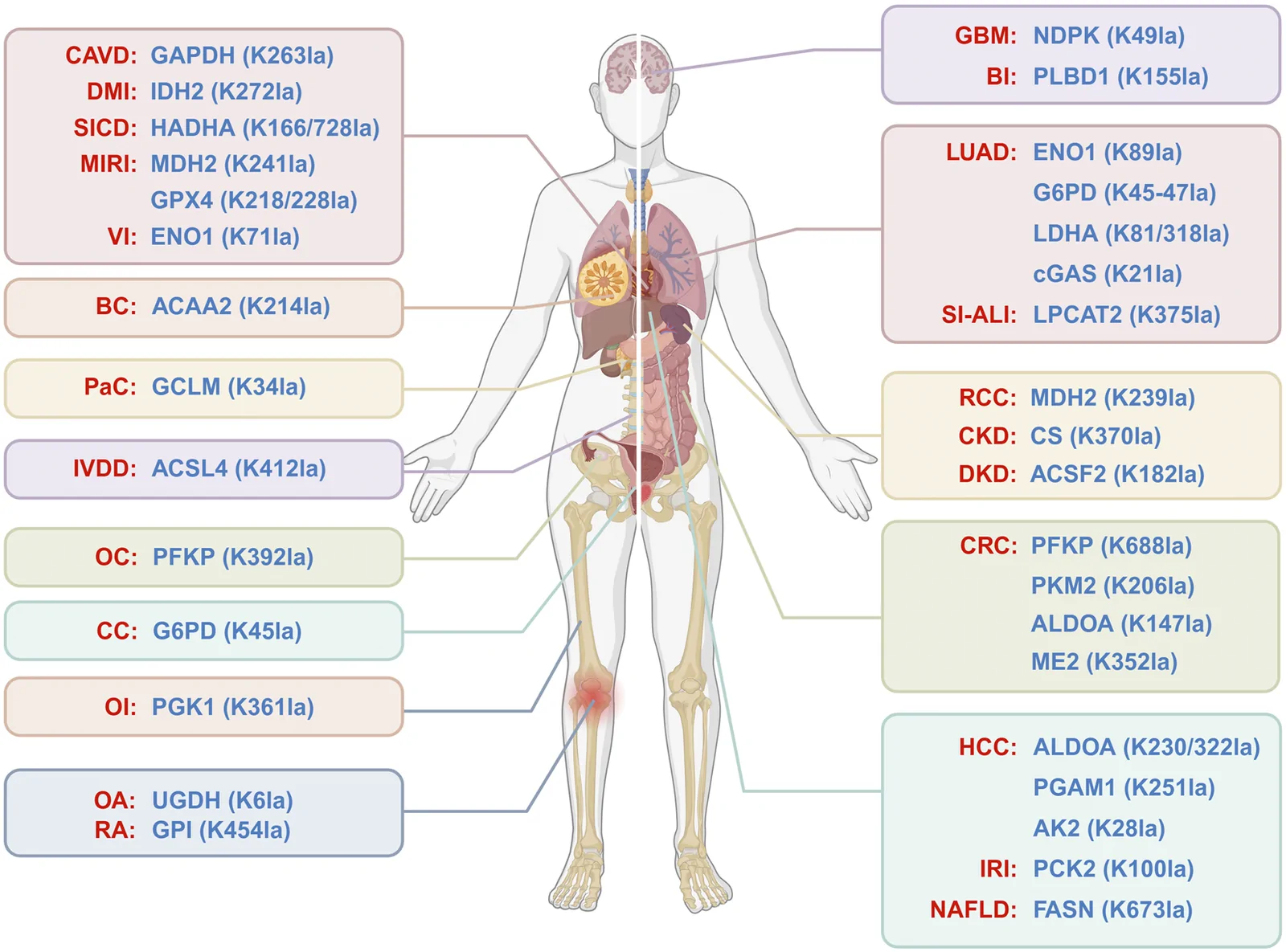
Metabolic enzyme lactylation and human diseases. Protein lactylation influences the onset and progression of various diseases, including cancer, inflammation, tissue injury and organ damage, degenerative diseases, metabolic dysfunction-associated fatty liver disease, and others. IVDD, intervertebral disc degeneration; CAVD, calcific aortic valve disease; DMI, diabetic myocardial infarction; SICD, sepsis-induced cardiac dysfunction; MIRI, myocardial ischemia-reperfusion injury; VI, vascular injury; BC, breast cancer; PaC, pancreatic cancer; OC, ovarian cancer; CC, cervical cancer; OI, osseous injury; GBM, glioblastoma; BI, brain injury; LUAD, lung adenocarcinoma; SI-ALI, sepsis-induced acute lung injury; RCC, renal cell carcinoma; CKD, chronic kidney disease; DKD, diabetic kidney disease; CRC, colorectal cancer; HCC, hepatocellular carcinoma; IRI, ischemia-reperfusion injury; NAFLD, non-alcoholic fatty liver disease; OA, osteoarthritis; RA, rheumatoid arthritis. Created with BioRender.com.

Accumulating evidence has linked lactylation of metabolic enzymes to tumor progression across various cancer types (Figure 6), with context-dependent outcomes ranging from enhanced proliferation to modulated drug resistance. Due to space limitations, this section focuses solely on the most prevalent cancer types worldwide. Information on lactylation of metabolic enzymes identified in other cancer types is provided in Section 3 and Figure 6, and Table 1.

#### 6.1.1 Lung cancer

In lung adenocarcinoma, lactylation of metabolic enzymes is associated with tumor aggressiveness, therapeutic resistance, and unfavorable clinical outcomes. Among these modifications, ENO1 and LDHA lactylation are involved in metabolic adaptation and resistance to osimertinib or cisplatin, respectively (Gan et al., 2026; Li J. et al., 2026). G6PD lactylation is associated with enhanced malignant phenotypes, including increased proliferation and migration, whereas cGAS lactylation is linked to impaired immune function and poor prognosis, suggesting that lactylation may serve as an important regulator of lung adenocarcinoma progression (Rao et al., 2025; Zhang Y. et al., 2025).

#### 6.1.2 Breast cancer

In triple-negative breast cancer, LDHC4-mediated ACAA2 lactylation is associated with metabolic reprogramming and tumor progression, suggesting a potential contribution of metabolic enzyme lactylation to the development of triple-negative breast cancer (Cui et al., 2025).

#### 6.1.3 Hepatocellular carcinoma

In hepatocellular carcinoma, lactylation of metabolic enzymes has been associated with malignant progression, including enhanced cancer cell proliferation, stemness, and metastatic potential. ALDOA, PGAM1, and mitochondrial AK2 lactylation represent potential mechanisms linking metabolic enzyme regulation to hepatocellular carcinoma development and progression (Yang Z. et al., 2023; Feng et al., 2024; Li Q. et al., 2026).

#### 6.1.4 Colorectal cancer

In colorectal cancer, lactylation of metabolic enzymes has emerged as a potential regulator of tumor progression and therapeutic resistance. PKM2 and ME2 lactylation are associated with enhanced malignant phenotypes, including vascular mimicry, drug resistance, and tumor cell proliferation, whereas PFKP and ALDOA lactylation have been identified in colorectal cancer cells with their functional roles requiring further investigation (Cheng et al., 2024; Li C. et al., 2025; Li W. et al., 2026).

Collectively, these findings demonstrate that lactylation of metabolic enzymes regulates tumor progression across diverse cancer types, often in a context- and site-specific manner, providing potential targets for therapeutic intervention.

### 6.2 Lactylation of metabolic enzymes in inflammatory responses

Lactylation of metabolic enzymes also plays a role in modulating inflammatory responses, with effects that vary depending on the specific enzyme and cellular context (Figure 6). In inflammatory diseases, lactylation of metabolic enzymes has emerged as an important regulator of disease progression by reshaping cellular metabolism and inflammatory responses. In macrophage-mediated inflammation, PKM2 lactylation contributes to metabolic reprogramming and promotes an anti-inflammatory phenotype (Wang et al., 2022). In interstitial kidney inflammation, osteoarthritis, and rheumatoid arthritis, lactylation of metabolic enzymes such as CS, UGDH, ACLY, and GPI is associated with enhanced inflammatory responses, tissue damage, and disease progression, highlighting the potential role of metabolic enzyme lactylation in inflammatory disease regulation (Zhang Y. et al., 2024; Lan et al., 2025; Tan et al., 2025; Yu et al., 2025).

### 6.3 Lactylation of metabolic enzymes in cardiovascular diseases

Lactylation of metabolic enzymes has emerged as a significant contributor to various cardiovascular pathologies, ranging from valve disease and endothelial dysfunction to myocardial injury (Figure 6). In cardiovascular diseases, lactylation of metabolic enzymes has been implicated in disease progression through diverse pathological processes. In calcific aortic valve disease, GAPDH lactylation contributes to disease development, whereas ENO1 and HADHA lactylation are associated with endothelial dysfunction and cardiac metabolic impairment during sepsis (Wang C. et al., 2025; Zhang T. N. et al., 2025; Xie et al., 2026). In diabetic myocardial infarction, IDH2 lactylation is linked to improved angiogenic responses and disease outcomes (Zang et al., 2025). Furthermore, MDH2 and GPX4 lactylation contribute to myocardial ischemia-reperfusion injury by promoting mitochondrial dysfunction and ferroptosis, respectively, highlighting the involvement of metabolic enzyme lactylation in cardiovascular disease progression (She et al., 2024; Wang Y. et al., 2025).

### 6.4 Lactylation of metabolic enzymes in other diseases

#### 6.4.1 Tissue injury and organ damage

Lactylation of metabolic enzymes has emerged as a potential regulator of tissue injury across multiple organs. In ischemic stroke, PLBD1 lactylation is associated with neuronal damage and neurological dysfunction (Zhou F. et al., 2025). In bone infection, PGK1 lactylation contributes to mitochondrial impairment and ferroptotic damage, thereby promoting bone tissue destruction (Qin et al., 2026). Moreover, LPCAT2 and PCK2 lactylation are involved in the progression of sepsis-induced lung injury and liver ischemia-reperfusion injury, respectively, through metabolic dysregulation and ferroptosis (Yuan et al., 2025; Deng et al., 2026). These findings highlight the potential role of metabolic enzyme lactylation in tissue injury and organ dysfunction.

#### 6.4.2 Degenerative diseases

In intervertebral disc degeneration, lactylation of metabolic enzymes has been implicated in disease progression through the regulation of oxidative stress and cell death pathways. In intervertebral disc degeneration, ACSL4-associated lactylation events contribute to nucleus pulposus cell dysfunction and tissue degeneration (Sun K. et al., 2025). In diabetic nephropathy, ACSF2 lactylation is associated with mitochondrial dysfunction and oxidative damage under hyperglycemic conditions, highlighting the potential role of metabolic enzyme lactylation in chronic disease development (Chen et al., 2024a).

#### 6.4.3 Metabolic dysfunction-associated fatty liver disease

In non-alcoholic fatty liver disease, MPC1-dependent regulation of FASN lactylation contributes to hepatic lipid homeostasis and disease progression. Reduced FASN lactylation favors lipid accumulation, whereas enhanced lactylation suppresses hepatic fat synthesis, suggesting that metabolic enzyme lactylation may represent an important link between metabolic remodeling and fatty liver disease development (Gao et al., 2023).

#### 6.4.4 PE

In PE, altered lactylation patterns in placental trophoblasts have been associated with impaired placental function and disease development. HK2 lactylation is involved in regulating trophoblast proliferation, and its disruption under oxidative stress may contribute to PE pathogenesis, highlighting the potential role of metabolic enzyme lactylation in pregnancy-related disorders (Chen Z. et al., 2025).

## 7 Lactylation modulates metabolic enzymes via histone-mediated transcription and indirect effects through lactylation of non-metabolic proteins

Beyond direct lactylation of metabolic enzymes, lactylation modulates metabolism via two indirect routes: histone lactylation epigenetically regulates metabolic enzyme transcription, and lactylation of non-histone proteins affects metabolic enzyme expression or activity. Notably, these regulatory routes vary across different metabolic pathways, reflecting a diverse array of lactylation-mediated control mechanisms.

### 7.1 Indirect regulation of glucose metabolism via lactylation

Histone lactylation promotes glycolysis by epigenetically upregulating the transcription of key glycolytic enzymes. A recent study observed that enhanced H3K18la was enriched at the promoters of glucose metabolism genes and upregulated *HK2* transcription, thereby driving liver macrophage M1 polarization and contributing to MASLD (Li J. et al., 2025). Another study has shown that H4K79la and H4K91la enhance the transcription of *LDHA*, *PGK1*, and *HK1*, thereby forming a feedback loop that promotes breast cancer progression (Liu J. et al., 2025). Histone lactylation has also been shown to promote the transcription of glycolytic enzyme genes, including *PKM*, leading to metabolic dysregulation and poor prognosis in non-small cell lung cancer (Jiang et al., 2021). Furthermore, studies have found that H4K12la promotes PKM2 expression, thereby driving the progression of AD (Pan et al., 2022).

Beyond histone-mediated transcriptional regulation of metabolic enzymes, Wei et al. further reported that H3K18la promoted USP39 expression, and USP39 extended PGK1 half-life through its deubiquitinating activity, thereby enhancing glycolysis and accelerating tumor progression (Wei et al., 2024). Non-histone proteins can also undergo lactylation to regulate metabolic enzymes involved in glucose metabolism. For instance, a study revealed that lactylation of discoidin, CUB, and LCCL domain-containing 1 (DCBLD1) at K172 increased its stability, which in turn promoted G6PD activation, ultimately accelerating cervical cancer progression (Meng et al., 2024a).

### 7.2 Indirect regulation of lipid metabolism via lactylation

Emerging evidence has established H3K18la as the most extensively characterized histone lactylation site in the regulation of lipid metabolism. In pancreatic cancer, a recent study showed that H3K18la promoted ACLY transcription, thereby promoting fatty acid synthesis and intracellular lipid accumulation, which subsequently drove cancer progression (Lu et al., 2025). Similarly, another study has demonstrated that H3K18la promotes the transcription of *elongation of very long chain fatty acids protein 5* (*ELOVL5*), induces lipid peroxidation, and facilitates ferroptosis (Hong J. et al., 2025). In liver cells, elevated H3K18la activated the CD36-NLRP3 inflammasome axis, promoting lipid accumulation and inflammation (Li H. et al., 2025). In intervertebral disc degeneration and primary mouse pulmonary microvascular endothelial cells, lactate-driven H3K18la promoted *ACSL4* transcription, thereby inducing ferroptosis (Fang et al., 2025; Sun K. et al., 2025). In cervical cancer, H3K18la promoted *GPD2* transcription, increased GPD2 expression, and consequently drove M2 macrophage polarization, contributing to malignant progression (Huang C. et al., 2024).

Beyond fatty acid metabolism, H3K18la also impacted cholesterol biosynthesis. A recent study reported that it promoted *acetyl-CoA acetyltransferase 2* (*ACAT2*) transcription and enhanced cholesterol secretion via small extracellular vesicles, thereby inducing M2 polarization of tumor-associated macrophages and establishing an immunosuppressive tumor microenvironment (Yang Y. et al., 2025). H3K18la further drove steroid hormone production by promoting the transcription of *cytochrome P450 family 11 subfamily A member 1* (*CYP11A1*) (Wu et al., 2024).

H4K16la has also been implicated in metabolic regulation. One study revealed that it activated the transcription of *pyruvate dehydrogenase kinase 4* (*PDK4*). Subsequently, it formed a lactate-H4K16la-PDK4 feedback loop that drove metabolic reprogramming, promoting lactate and lipid accumulation, as well as liver damage (Jiao et al., 2025).

In addition to direct transcriptional regulation, lactylation of non-metabolic proteins can indirectly reshape lipid metabolism. Du et al. demonstrated that H3K18la promoted YTHDC1 expression, an m6A reader that stabilized m6A-modified nuclear enriched abundant transcript 1 (NEAT1). NEAT1 then recruited p300 to enhance histone acetylation at the stearoyl-CoA desaturase (SCD) promoter, thereby driving lipid metabolic reprogramming and hepatocellular carcinoma progression (Du et al., 2024). A recent study showed that H3K14la promoted the PARK7-fatty acid desaturase 1/2 (FADS1/2) axis, through which FADS1/2 promoted long-chain polyunsaturated fatty acid (PUFA) metabolism, conferring resistance to ferroptosis and alleviating acute lung injury (Xu J. et al., 2025).

Non-histone lactylation has also been implicated in lipid metabolism. A recent study reported that apolipoprotein C-II (APOC2) underwent K70 lactylation, enhancing its interaction with lipoprotein lipase, thereby promoting free fatty acid release and immunotherapy resistance in non-small cell lung cancer (Chen et al., 2024b). In neuronal protection, lactylation of methyl-CpG binding protein 2 (MECP2) at K210 and K249 inhibited *PLA2G6* transcription, an enzyme that hydrolyzes glycerophospholipids to release free fatty acids and lysophospholipids, thereby attenuating neuronal apoptosis and preventing ischemic brain injury (Sun M. et al., 2025).

Lactylation dynamics also impacted cholesterol metabolism. Zinc-finger MIZ-type-containing protein 1 (ZMIZ1) underwent K843 lactylation, which enhanced its binding to *Nanog homeobox* (*NANOG*), increasing *NANOG* transcriptional activity. NANOG subsequently promoted the expression of Niemann-Pick disease type C2 (NPC2), thereby enhancing cholesterol uptake, cancer stemness, and tamoxifen resistance in breast cancer (Liu et al., 2025c). Wang et al. demonstrated that HDAC2-dependent PD-L1 K189 delactylation promoted its nuclear localization and binding to the transcription factor YY1, thereby increasing *SQLE* transcription, promoting cholesterol biosynthesis, and facilitating liver cancer growth (Wang X. et al., 2025).

### 7.3 Indirect regulation of amino acid metabolism via lactylation

Histone lactylation has been shown to regulate amino acid metabolism by epigenetically modulating the transcription of key enzymes. A recent study by Huang et al. revealed that H3K18la modification enhances *cysteine desulfurase* (*NFS1*) transcription. NFS1 upregulation promotes iron-sulfur (Fe-S) cluster biosynthesis, thereby reducing ferroptosis susceptibility and driving hepatocellular carcinoma metastasis following insufficient microwave ablation (Huang et al., 2025). She and colleagues demonstrated that H3K18la promotes arginase 1 (ARG1) expression, leading to mitochondrial dysfunction and PANoptosis in ischemia/hypoxia-induced vascular dysfunction. Although ARG1 is a key enzyme in converting arginine to urea and ornithine, its role in the urea cycle was not addressed (She et al., 2025).

Lactylation of non-histone proteins also indirectly modulates amino acid metabolism. For instance, p300-mediated YY1 K183 lactylation was shown to promote IDO1 expression in tryptophan metabolism, thereby aggravating autoimmune uveitis (Huang J. et al., 2024). Furthermore, Lu et al. identified that, in lenvatinib-resistant hepatocellular carcinoma models, insulin-like growth factor 2 mRNA-binding protein 3 (IGF2BP3) K76 lactylation enhanced its binding to *PCK2* and *nuclear factor erythroid 2-related factor 2* (*NRF2*) mRNAs, promoting their expression. This modification reprogrammed serine metabolism, and the resulting increase in S-adenosylmethionine drove m6A methylation of *PCK2* and *NRF2* mRNAs, forming a positive feedback loop that sustained their expression and lenvatinib resistance (Lu et al., 2024).

### 7.4 Indirect regulation of nucleotide metabolism via lactylation

Compared with glucose and lipid metabolism, evidence for indirect regulation of nucleotide metabolism via lactylation remains limited. A recent study demonstrated that H3K18la promoted *Ras homolog enriched in striatum* (*RASD2*) transcription. Subsequently, RASD2 enhanced the stability of the pyrimidine metabolic enzyme CTP synthase 1 (CTPS1) by promoting its SUMOylation and inhibiting its ubiquitination, thereby contributing to endometriosis progression (Wang Z. et al., 2025).

## 8 Conclusion and perspectives

Since the discovery of protein lactylation in 2019 (Zhang et al., 2019), this lactate-derived post-translational modification has emerged as a cutting-edge research frontier in biology. Lactylation alters protein conformation, charge, and molecular interactions, thereby modulating key protein properties including enzymatic activity, stability, subcellular localization, and binding capacity. At the molecular level, these changes enable lactylation to regulate a broad spectrum of biological processes, such as gene expression, cellular metabolism, signal transduction, and programmed cell death. Consequently, lactylation is implicated in the pathogenesis of various diseases, including cancer, inflammatory disorders, cardiovascular diseases, and neurodegenerative diseases (Figure 6).

In this review, we systematically summarized the reciprocal metabolic–lactylation feedback loop and highlighted how lactylation regulates metabolic enzyme properties, including catalytic activity, protein stability, and molecular interactions (Figure 5; Table 1). A recurring theme is that lactylation exerts bidirectional effects on enzyme function—either activating or inhibiting—depending on the specific enzyme, the modified residue, and the cellular context. Notably, the same enzyme can exhibit opposing functional outcomes when lactylated at different sites (Cheng et al., 2024; Mi et al., 2025; Gan et al., 2026; Xie et al., 2026). These observations underscore the importance of site-specific analysis in future studies.

Previous analysis revealed that the number of lactylated proteins was far lower in normal cells than in tumor cells (Yang Y. H. et al., 2023), suggesting that aberrant lactylation may serve as a hallmark of metabolic rewiring in cancer—a notion consistent with the Warburg effect. This observation points to a bidirectional interplay between metabolic enzymes and lactylation. On one hand, metabolic enzymes control lactate production and thereby shape the lactylation landscape. On the other hand, lactylation exerts feedback regulation through two mechanisms. Directly, lactylation of metabolic enzymes *per se* alters their intrinsic properties, including activity, stability, and interactions. Indirectly, lactylation modulates the expression or activity of metabolic enzymes via two routes: histone lactylation epigenetically regulates the transcription of metabolic enzymes, while lactylation of other proteins affects the expression or activity of metabolic enzymes. Mass spectrometry-based identification across several tumor types has revealed eight metabolic enzymes that undergo lactylation in multiple cancers, six of which are closely linked to glucose metabolism. Beyond glucose metabolism, enzymes involved in lipid, nucleotide, and amino acid metabolism are also highly lactylated, further emphasizing the pervasive interplay between this modification and cellular metabolism.

Despite these advances, several fundamental questions remain unanswered:

- Beyond Activity and Stability: Multi-dimensional Regulation of Metabolic Enzymes by Lactylation

Current research has predominantly focused on how lactylation affects the catalytic activity and protein stability of metabolic enzymes. However, lactylation may also regulate metabolic enzymes through additional mechanisms, including altering subcellular localization, modulating substrate specificity, and affecting interactions with other metabolic enzymes or signaling molecules. Systematic investigation of these multi-dimensional regulatory effects will be essential for a comprehensive understanding of how lactylation controls metabolic enzyme function.

- Dynamic Regulation of Metabolic Enzyme Lactylation in Response to Metabolic States

Metabolic enzymes both control lactate production and serve as substrates for lactylation, creating a potential feedback circuit. How metabolic enzyme lactylation dynamically changes under different metabolic states—such as glycolysis-dominant versus oxidative phosphorylation-dominant conditions—remains unclear. Similarly, whether metabolic enzymes in distinct subcellular compartments exhibit differential lactylation patterns warrants investigation. Development of sensitive detection tools, including lactylation site-specific antibodies, will be critical for addressing these questions.

- Limitations in Understanding the Specificity of Lactylation Writers and Erasers

Although previous studies have reported that several proteins exhibit lactylation “writer” or “eraser” activities, the molecular mechanisms governing lactylation regulation and the enzymatic specificity of these regulatory factors remain incompletely understood. In particular, their substrate preferences, the experimental evidence supporting site-specific regulation, and the extent to which their activities are influenced by cellular metabolic states and microenvironmental conditions require further investigation. Moreover, whether protein lactylation is regulated in a compartment-specific manner according to subcellular localization or local metabolic environments remains largely unexplored. Future studies should focus on systematically defining the substrate selectivity of lactylation writers and erasers, while developing precise site-specific detection and editing technologies to further elucidate their regulatory mechanisms and biological functions.

- Crosstalk Between Lactylation and Other Post-Translational Modifications on Metabolic Enzymes

Metabolic enzymes are regulated by diverse PTMs, including acetylation, succinylation, and ubiquitination. As a recently identified PTM, lactylation remains less understood in terms of its molecular mechanisms and structural basis. Emerging evidence suggests that lactylation may crosstalk with other PTMs; for example, it may compete with ubiquitination for lysine residues. Given that many lactylation regulators also control acetylation, potential competition between lactylation and acetylation may exist. Moreover, the reported generation of lactyl-CoA and succinyl-CoA by GTPSCS suggests possible crosstalk between lactylation and succinylation. Future studies should further define these PTM interactions.

- *In vivo* Functional Validation: The Scarcity of Metabolic Enzyme Lactylation Knockin Mouse Models

The vast majority of lactylation functional studies rely on cell-based systems or *in vitro* experiments, leaving the *in vivo* physiological and pathological significance of metabolic enzyme lactylation largely undefined. Systematic generation of lactylation-deficient or lactylation-mimetic knockin mice targeting specific metabolic enzyme sites is urgently needed.

- Large-Scale Lactylomics Analysis: Systematic Comparison of Metabolic Enzyme Lactylation Profiles Between Tumor and Normal Tissues

Lactylomics studies have identified thousands of lactylation sites across multiple cancer types, with metabolic enzymes consistently enriched among the modified proteins. However, systematic comparisons of metabolic enzyme lactylation profiles between tumor and normal tissues remain in their early stages. Future efforts should focus on constructing comprehensive “tumor metabolic enzyme lactylation atlases” to identify cancer-specific lactylation events with diagnostic or therapeutic potential.

- Methodological Limitations of Current Research Methods for Lactylation

Current lactylation research faces several challenges, including limited antibody specificity, insufficient discrimination between D- and L-lactylation by mass spectrometry, and inadequate validation of modification sites. Moreover, the lack of site-specific editing tools and structural analyses limits our understanding of how lactylation regulates protein function. Current studies mainly rely on K→Q mutations or computational predictions, whereas direct structural evidence remains scarce. Future research should integrate advanced detection and editing technologies with high-resolution approaches, such as cryo-electron microscopy and X-ray crystallography, to define lactylation sites and elucidate their effects on protein conformation, interactions, and enzymatic activity.

- Context-Dependent Roles of Lactylation in Disease Progression

Due to the increasing number of studies investigating the association between lactylation and disease, lactylation is often perceived as a disease-promoting modification. However, lactylation is not exclusively associated with pathological progression and may exert context-dependent effects. In cancer, the Warburg effect leads to elevated intracellular lactate levels, which can increase global lactylation and potentially reflect a metabolic adaptation response of tumor cells. Nevertheless, increased lactylation does not necessarily promote tumor progression, and certain lactylation events may even exert tumor-suppressive effects. Further studies are required to elucidate the diverse and context-specific roles of lactylation in disease development.

- Therapeutic Strategies Targeting Metabolic Enzyme Lactylation

Given that lactylation levels are significantly elevated in tumor cells and contribute to metabolic reprogramming and chemotherapy resistance, targeting specific lactylation events on metabolic enzymes may represent a novel therapeutic strategy. Structure-based inhibitors or PROTAC technology could be developed to target aberrantly lactylated metabolic enzymes.

In summary, lactylation of metabolic enzymes represents a rapidly growing field that bridges metabolism and post-translational regulation. A deeper understanding of its mechanisms and functions will not only illuminate fundamental biological principles but also open new avenues for therapeutic intervention in cancer, inflammatory diseases, and beyond.

## Glossary

LDH: lactate dehydrogenase
MCTs: monocarboxylate transporters
Kla: lysine lactylation
**K**_**L-la**_: lysine L-lactylation
**K**_**D-la**_: lysine D-lactylation
**K**_**ce**_: N-ε-(carboxyethyl)-lysine
LDHA: lactate dehydrogenase A
LDHB: lactate dehydrogenase B
MGO: methylglyoxal
LGSH: lactoylglutathione
GSH: glutathione
GLO1: glyoxalase 1
KAT/HAT: lysine acetyltransferase
AARS1/2: alanyl-tRNA synthetase 1/2
CBP: CREB-binding protein
GCN5/KAT2A: general control non-derepressible 5
PCAF: p300/CBP-associated factor
TIP60/KAT5: Tat-interactive protein 60
HBO1/KAT7: histone acetyltransferase binding to ORC1
MOF/KAT8: males absent on the first
YTHDC1: YTH domain-containing protein 1
VEGF-A: vascular endothelial growth factor A
PDHA1: pyruvate dehydrogenase E1 subunit alpha 1
CPT2: carnitine palmitoyltransferase 2
cGAS: cyclic GMP-AMP synthase
p53: tumor protein
HDAC6: histone deacetylase 6
ACSS2: acetyl-CoA synthetase 2
ERK: extracellular signal-regulated kinase
CoA: coenzyme A
GTPSCS: guanosine triphosphate-specific succinyl-CoA synthetase
OXPHOS: oxidative phosphorylation
SIRT: sirtuin
METTL3: N6-methyltransferase-like 3
PRMT1: protein arginine methyltransferase 1
ME2: malic enzyme 2
ROS: reactive oxygen species
BRG1: brahma-related gene 1
TRIM33: tripartite motif-containing protein 33
DPF2: double PHD fingers 2
PRMT5: protein arginine methyltransferase 5
ALKBH5: alkB homolog 5
TFEB: transcription factor EB
WWP2: WW domain-containing E3 ubiquitin protein ligase 2
METTL16: methyltransferase-like 16
FDX1: ferredoxin 1
CD44: cluster of differentiation 44
RIG-I: retinoic acid-inducible gene I protein
PARP1: poly(ADP-ribose) polymerase 1
PKM2: pyruvate kinase M2
FOSL1: fos-related antigen 1
EGR1: early growth response 1
PFK: phosphofructokinase
PFKM: phosphofructokinase M
PFKP: phosphofructokinase P
ZEB1: zinc finger E-box-binding homeobox 1
CNTN1: contactin 1
ENO2: enolase 2
TPI1: triosephosphate isomerase 1
EMT: epithelial-mesenchymal transition
ENO1: enolase 1
GDF15: growth differentiation factor 15
IDH3β: isocitrate dehydrogenase 3β
TCA: tricarboxylic acid
AD: Alzheimer’s disease
PAX6: paired-box gene 6
ALDOB: aldolase B
PDK1: pyruvate dehydrogenase kinase-1
CEACAM6: carcinoembryonic antigen cell adhesion molecule 6
GAPDH: glyceraldehyde-3-phosphate dehydrogenase
SNAIL1: snail family transcriptional repressor 1
FIS1: mitochondrial fission 1 protein
PCK1: phosphoenolpyruvate carboxykinase 1
SPRING: sterol regulatory element-binding protein (SREBP) regulating gene
ALDOA: aldolase A
PYGL: glycogen phosphorylase L
ADK: adenosine kinase
PRDX1: peroxiredoxin 1
ABCF1: ATP binding cassette subfamily F member 1
HK2: hexokinase 2
VDAC1: voltage-dependent anion channel 1
GPI: glucose-6-phosphate isomerase
PTEN: phosphatase and tensin homolog
PGK1: phosphoglycerate kinase 1
VDAC3: voltage-dependent anion channel 3
PGAM1: phosphoglycerate mutase 1
PKM1: pyruvate kinase M1
MDH2: malate dehydrogenase 2
CNOT6: CCR4-NOT transcription complex subunit 6
VE-cadherin: vascular endothelial cadherin
CS: citrate synthase
IDH2: isocitrate dehydrogenase 2
NLRP3: NOD-, LRR- and pyrin domain-containing protein 3
eNOS: endothelial nitric oxide synthase
SLC25A1: citrate transporter solute carrier family 25 member 1
PC: pyruvate carboxylase
PCK2: phosphoenolpyruvate carboxykinase 2
OXSM: 3-oxoacyl-ACP synthase, mitochondrial
G6PD: glucose-6-phosphate dehydrogenase
HPV16 E6: human papillomavirus type 16 early protein 6
6PGD: 6-phosphogluconate dehydrogenase
TKT: transketolase
UGDH: UDP-glucose dehydrogenase
STAT1: signal transducer and activator of transcription 1
MAP3K8: mitogen-activated protein kinase kinase kinase 8
HSL: hormone-sensitive lipase
PLBD1: phospholipase B domain-containing protein 1
ACS: acyl-CoA synthetase
ACSL1: acyl-CoA synthetase long-chain family member 1
ACSF2: acyl-CoA synthetase family member 2
DKD: diabetic kidney disease
HADHA: hydroxyacyl-CoA dehydrogenase trifunctional multienzyme complex subunit alpha
ACAA2: acetyl-CoA acyltransferase 2
LDHC4: lactate dehydrogenase C4
ACLY: ATP-citrate lyase
Acly L: Acly Long
Acly S: Acly Short
RBM25: RNA-binding motif protein 25
FASN: fatty acid synthase
LPCAT2: lysophosphatidylcholine acyltransferase 2
GPX4: glutathione peroxidase 4
VPS34: vacuolar protein sorting 34
H/R: hypoxia/reoxygenation
PI3P: phosphatidylinositol 3-phosphate
ATG14L: autophagy-related 14-like protein
UVRAG: UV radiation resistance-associated gene
GCL: glutamate-cysteine ligase
GCLM: glutamate-cysteine ligase M
PHGDH: phosphoglycerate dehydrogenase
NDPK: nucleoside diphosphate kinase
AK2: adenylate kinase 2
PE: preeclampsia
LCSCs: liver cancer stem cells
DCBLD1: discoidin, CUB, and LCCL domain-containing 1
ELOVL5: elongation of very long chain fatty acids protein 5
PUFA: long-chain polyunsaturated fatty acid
ACAT2: acetyl-CoA acetyltransferase 2
CYP11A1: cytochrome P450 family 11 subfamily A member 1
PDK4: pyruvate dehydrogenase kinase 4
NEAT1: nuclear enriched abundant transcript 1
SCD: stearoyl-CoA desaturase
FADS1/2: fatty acid desaturase 1/2
APOC2: apolipoprotein C-II
MECP2: methyl-CpG binding protein 2
ZMIZ1: zinc-finger MIZ-type-containing protein 1
NANOG: Nanog homeobox
NPC2: niemann-pick disease type C2
NFS1: cysteine desulfurase
Fe-S: iron-sulfur
ARG1: arginase 1
IGF2BP3: insulin-like growth factor 2 mRNA-binding protein 3
NRF2: nuclear factor erythroid 2-related factor 2
RASD2: Ras homolog enriched in striatum
CTPS1: CTP synthase 1
